# Untargeted Metabolomics Reveals Dose-Response Characteristics for Effect of Rhubarb in a Rat Model of Cholestasis

**DOI:** 10.3389/fphar.2016.00085

**Published:** 2016-03-31

**Authors:** Cong-En Zhang, Ming Niu, Rui-Yu Li, Wu-Wen Feng, Xiao Ma, Qin Dong, Zhi-Jie Ma, Guang-Quan Li, Ya-Kun Meng, Ya Wang, Ping Yin, Lan-Zhi He, Yu-Meng Li, Peng Tan, Yan-Ling Zhao, Jia-Bo Wang, Xiao-Ping Dong, Xiao-He Xiao

**Affiliations:** ^1^College of Pharmacy, Chengdu University of Traditional Chinese MedicineChengdu, China; ^2^China Military Institute of Chinese Medicine, 302 Military HospitalBeijing, China; ^3^Department of Pharmacy, Beijing Friendship Hospital, Capital Medical UniversityBeijing, China

**Keywords:** metabolomics, cholestasis, rhubarb, dose response, pathway analysis

## Abstract

Cholestasis is a serious manifestation of liver diseases with limited therapies. Rhubarb, a widely used herbal medicine, has been frequently used at a relatively large dose for treating cholestasis. However, whether large doses are optimal and the therapeutic mechanism remain unclear. To explore these questions, the anti-cholestatic effect of five doses of rhubarb (0.21, 0.66, 2.10, 6.60, and 21.0 g/kg) in an alpha-naphthylisothiocyanate (ANIT)-induced rat model of cholestasis was examined by histopathology and serum biochemistry. A dose-dependent anti-cholestatic effect of rhubarb (0.21–6.6 g/kg) was observed, and an overdose of 21.0 g/kg showed a poor effect. LC-MS-based untargeted metabolomics together with pathway analysis were further applied to characterize the metabolic alterations induced by the different rhubarb doses. Altogether, 13 biomarkers were identified. The dose-response curve based on nine important biomarkers indicated that doses in the 0.42–6.61 g/kg range (EC_20_–EC_80_ range, corresponding to 4.00–62.95 g in the clinic) were effective for cholestasis treatment. The pathway analysis showed that bile acid metabolism and excretion, inflammation and amino acid metabolism were altered by rhubarb in a dose-dependent manner and might be involved in the dose-response relationship and therapeutic mechanism of rhubarb for cholestasis treatment.

## Introduction

Cholestasis is one of the most common and devastating manifestations of liver diseases. The interrupted excretion of bile due to functional disorders of bile-producing cells or a mechanical block along the course of the biliary channels outside of the liver can lead to cholestasis (Trauner et al., 1998; Ghonem et al., 2015). Although the importance of the disease has been promoted, more research is needed for the complete understanding of cholestasis. Additionally, only limited therapies are available for this disease (Bacq et al., 2012; Beuers et al., 2015).

At present, ursodeoxycholic acid (UDCA) is the only widely used drug to treat cholestatic diseases. However, UDCA does not provide consistent efficacy, and some patients do not respond to UDCA (Beuers, 2006; Jones et al., 2010). Herbal medicine has a long history for the treatment of cholestasis (Ghosh et al., 2011). Rhubarb (Rhei Radix et Rhizoma) is a famous medicine officially documented in the Chinese Pharmacopoeia (National Pharmacopoeia Committee, 2010), that has been used for the treatment of cholestasis since the time of the Eastern Hang dynasty (25–220 AD) as part of the Yin-Chen-Hao-Tang preparation (Wang et al., 2011b). Rhubarb has been shown to possess broad spectrum activities, such as antioxidant (Silveira et al., 2013), anti-inflammatory (Hu et al., 2014), and hepatoprotective activities (Neyrinck et al., 2014); moreover, rhubarb encourage bilirubin excretion (Sim et al., 2015), and has been applied for the treatment of cholestasis in the clinic (Ho, 1996; Huang et al., 1997).

To date, rhubarb has been frequently used at a relatively large dose to treat cholestasis (up to 60 g of crude drug per day), which is more than four times the regular dose recommended in the Chinese Pharmacopoeia (3–15 g). Some clinical cases and experimental studies have demonstrated its satisfactory therapeutic effects with no toxicity and side effects (Hu, 1986; Huang et al., 1997; Lv et al., 2011; Yang et al., 2012). However, the anti-cholestatic effect of rhubarb at a low dose is not well-documented, and little is known about the scientific connotation of the dose-response relationship of rhubarb for the treatment of cholestasis. Therefore, further study is needed to explore suitable doses of rhubarb and its therapeutic mechanism of action for the treatment of cholestasis. Because the various chemical constituents of rhubarb can simultaneously hit different therapeutic targets for cholestasis, studies on the dose-effect relationship of rhubarb for the treatment of cholestasis and its pharmacological mechanism of action are limited by the inability to measure a wide spectrum of potential biological changes.

Untargeted metabolomics has been widely applied to evaluate the therapeutic effects of herbal medicines due to their multiple components, multiple actions, and multiple targets (Wang et al., 2005, 2011c). Based on the analysis of specific early biomarkers and perturbed pathways during disease or drug treatment, untargeted metabolomics provides a holistic insight into the relationship between the substance and metabolic pathways that might clarify the mechanism of action (Suhre et al., 2011). Additionally, researchers have shown that alterations in the profiles of endogenous metabolites may parallel the development of cholestasis (Aoki et al., 2011; Wang et al., 2012; Long et al., 2015). Untargeted metabolomics has been successfully used to explore the therapeutic mechanisms of emodin (Ding et al., 2008), Yinchenhao (Sun et al., 2014), and the Huang-Lian-Jie-Du decoction (Wei et al., 2015) for the treatment of cholestasis.

α-naphthylisothiocyanate (ANIT) is a model hepatotoxin that induce reproducible hepatobiliary toxicity *in vivo* characterized by cessation of bile flow, hepatic parenchymal cell injury, and infiltration of neutrophils around bile ducts, which pathologically similar to cholestatic hepatitis. In experimental medicine, ANIT treatment has been extensively used, permitting to describe not only cholestatic alterations but also compensatory mechanisms (Padda et al., 2011). In the present study, five different doses of rhubarb were administered to an ANIT-induced rat model (Di Padova et al., 1985; Jean and Roth, 1995) of cholestasis. The anti-cholestatic effect of rhubarb was investigated using conventional approaches. To explore the dose-response relationship of rhubarb and its therapeutic mechanism of action for the treatment of cholestasis, a liquid chromatography-mass spectrometry (LC-MS)-based untargeted metabolomics approach was applied to characterize the metabolic alterations induced by different doses of rhubarb. Finally, a systematic analysis of specific biomarkers and unique biochemical pathways was conducted with multivariate data analysis techniques.

## Materials and methods

### Chemicals and reagents

Acetonitrile and methanol (HPLC grade) were purchased from Merck (Darmstadt, Germany) and Burdick and Jackson (Ulsan, Korea), respectively. Formic acid was purchased from Sigma-Aldrich Co. (St Louis, USA). Double-distilled water was purified by Millipore water purification system (Millipore, Bedford, MA). Other chemicals were of analytical grade. Rhubarb (*Rheum tanguticum* Maxim.ex Balf., Batch number R/20140423) collected from Ruoergai county, Sichuan province, China. The herb was authenticated by Dr. Xiao-He Xiao (Director, Military Institute of Chinese Materia Medica, Beijing, China). The decoction pieces of rhubarb were prepared with the aid of China Medico Corporation (Tianjin, China).

### Sample preparation and quality control of rhubarb

At first, the decoction pieces were added into boiling water, extracted twice, and 20 min each time. After the extraction was completed, the extract was filtrated and collected. The extract was concentrated by rotary evaporators under 60°C and dried to dryness in vacuum drier at 45°C. The final ratio of powder to raw herb was 28.82%. To ensure the quality of rhubarb used for animal studies. The content of main components was determined by using High Performance Liquid Chromatography (HPLC), and the chemical fingerprint of rhubarb was established. What's more, the chemical information of water extract was established by ultra-high performance liquid chromatography-mass spectrometry (UHPLC-MS) system. Full methods and any associated operational details are available in the Supplementary Materials.

### Animal handling and animal experiment design

A total of 80 male Sprague-Dawley rats weighing approximately 220–240 g were obtained from the Laboratory Animal Center of the Academy of Military Medical Sciences (Certification number SCXK-JUN 2007-004). The room temperature was regulated at 20 ± 2°C with 60–70% humidity. A 12-h light/dark cycle was set and the animals were provided free access to standard diet and water. All animals were acclimated for 7 days prior to the experiments.

**Figure 1:**
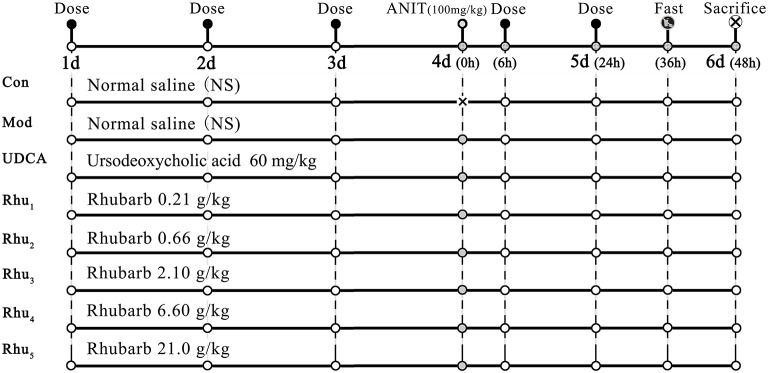
**The schematic diagram of the animal experimental design**. Con, control group treated with normal saline; Mod, model group treated with normal saline; UDCA, positive control group treated with ursodeoxycholic acid; Rhu_1_ – Rhu_5_, rats treated with rhubarb extract at doses of 0.21, 0.66, 2.10, 6.60, and 21.0 g/kg, respectively. × in the figure indicates rats without ANIT treatment.

Figure 1 shows the experimental design for the animal studies. The animals were randomly divided into eight groups with ten rats each as follows. Group (1) was the normal group and served as the control; the rats received normal saline each day and were treated with the vehicle (olive oil) alone. All other groups were intragastrically treated with 100 mg/kg ANIT in an equal volume of olive oil to establish the cholestasis animal model on day 4 (Di Padova et al., 1985; Jean and Roth, 1995; Zhao et al., 2009). Group (2) was the model group; except for the single dose of ANIT, the model group was treated with normal saline each day. Group (4)–Group (8) were the five rhubarb dose groups. The rhubarb water extract was dissolved in normal saline and intragastrically administered to the rats at doses of 0.21 (Group Rhu_1_), 0.66 (Group Rhu_2_), 2.10 (Group Rhu_3_), 6.60 (Group Rhu_4_), and 21.0 (Group Rhu_5_) g/kg, respectively, for 4 days before and 2 days after intragastric treatment with 100 mg/kg ANIT. Group (3) was the positive control UDCA group (60 mg/kg, Group UDCA) (Ding et al., 2008); the rats were treated with the same conditions as the rhubarb dosage groups. The animal doses of rhubarb corresponded to doses of 2.0, 6.3, 20, 63, and 200 g of rhubarb, respectively, (measured as the quantity of crude material) per day for a human weighing 60 kg (Xu et al., 1982).

After the last administration, the rats were provided a standard diet and water and then fasted for 12 h prior to sacrifice. The rats were sacrificed at 48 h after the administration of ANIT (on the sixth day) by intraperitoneal injection of pentobarbital sodium. Blood samples were collected from the inferior vena cava and the livers were removed from the rats immediately after sacrifice. Efforts were made to minimize animal suffering. The experimental protocol was approved by the Committee on the Ethics of Animal Experiments of the 302 Military Hospital.

### Serum biochemistry and histopathological analysis

Serum samples were obtained by separating the supernatant from the blood after 30 min of coagulation at room temperature. After centrifugation (3000 rpm for 10 min), the serum ALT, AST, total bilirubin (TBIL), direct bilirubin (DBIL), alkaline phosphatase (ALP), and total bile acid (TBA) levels were measured with an Olympus AU5400 automatic biochemistry analyzer (Olympus Optical, Tokyo, Japan). Liver tissues for histopathological examination were fixed and preserved in 10% neutral buffered formalin, processed and trimmed, embedded in paraffin, sectioned to a thickness of approximately 5 μm, and stained with hematoxylin and eosin (HE).

### Serum sample handling

Two hundred microliter thawed serum samples and 600 μL methanol were transferred to a 1.5 ml polypropylene tube, then the solution was mixed and allowed to stand for 20 min at 4°C before use. The samples were collected after centrifugation at 12,000 rpm for 10 min at 4°C to remove any solid debris, and the supernatant was transferred to a polypropylene tube, and then filtered through a syringe filter (0.22 μm), 4 μL of the supernatant were injected into the UHPLC-MS system.

### Chromatography and mass spectrometry conditions

The metabolic profiling analysis of the biofluids was conducted on an Agilent 6550 iFunnel Q-TOF LC/MS (Agilent Technologies, USA). The sample sequence was random and 4 μL aliquot of each sample was injected onto a ZORBOX RRHD C18 analytical column (2.1 mm i.d. × 100 mm, 1.7 μm i.d., Agilent Technologies, USA), the column temperature was maintained at 30°C. For the ESI+ analysis, separation was achieved with a 25 min linear gradient with the mobile phases of solvent A (Acetonitrile spiked with 0.1% formic acid) and solvent B (Water spiked with 0.1% formic acid). The flow rate was set as 0.30 mL/min. The gradient was used as follows: a linear gradient of 5% A over initial-1.0 min, 5–40% A over 1.0–9.0 min, 40–90% A over 9.0–19.0 min, 90–100% A over 19.0–21.0 min, 100% A over 21.0–25.0 min. The eluent was introduced to the mass spectrometer directly. To ensure the stability and repeatability of the systems, 10 μL of each sample were pooled as a quality control (QC) sample, which inserted and analyzed in every 10 samples. For the ESI− analysis, the mobile phases used were (A) acetonitrile and (B) water. The other analysis conditions were identical to ESI+ analysis.

For mass spectrometry, the Agilent 6550 Q-TOF/MS with an electrospray ionization source (ESI) in both positive and negative mode was used. The combination use of both positive and negative ionization LC-MS offers more comprehensive metabolome coverage than the use of a single polarity (Lei et al., 2011). The electrospray source parameters were fixed as follows: electrospray capillary voltage was 3.0 kV in negative ionization mode and 4.0 kV in positive ionization mode. The mass range was set from m/z 80 to 1000. Gas temperature was 200°C in negative ionization mode and 225°C in positive ionization mode. Gas flow was 11 L/min. Nebulizer was set to 35 pisg (negative) and 45 pisg (positive). Sheath gas temperature was 350°C and sheath gas flow was 12 L/min. Nozzle voltage was 500 V in both negative and positive mode. For internal mass calibration during the MS analysis, reference masses 121.0509 (Purine, [C_5_H_4_N_4_+ H] ^+^) and 922.0098 (HP-0921, [C_18_H_18_O_6_N_3_P_3_F_24_+ H] ^+^) were used in positive mode, and 112.9856 (TFANH_4_, [C_2_H_4_O_2_NF_3_− NH_4_]−) and 1033.9881 TFANH_4_ + HP-0921, [C_20_H_22_O_8_N_4_P_3_F_27_− NH_4_]−) were used in negative mode.

### Data processing and pattern recognition analysis

All data were pre-processed with Profinder (version B.06.00, Agilent Technologies, USA). For molecular feature extraction, up to 2000 compounds with a peak height above 300 counts were extracted. The initial and final retention times were set for data collection. The missing value estimation, data filtering and data normalization were achieved by the MetaboAlalyst 3.0 online software. The resultant data matrices were introduced into the SIMCA-P+ 13.0 (Umetrics, Umeå, Sweden) software for multivariate statistical analysis including principal component analysis (PCA) and orthogonal partial least-squares discriminant analysis (OPLS-DA). Prior to PCA, all variables obtained from the data matrix were mean-centered and scaled to the pareto variance. The PCA score plot was used to present the natural interrelationship among observations. To find potential biomarkers, the OPLS-DA model was used to explore deep differences between the control, model and rhubarb-treated groups. Variables with a VIP value (VIP ≥1.0) and |p(corr)| ≥ 0.5 (Wheelock and Wheelock, 2013) in the OPLS-DA model were selected as potential biomarkers.

### Biomarker identification and metabolic pathway analysis

Fold changes were calculated by the MetaboAnalyst 3.0 software. The significant differences were determined using the fold change value (> 1.5) combined with the ANOVA (*p* < 0.05) and *t*-test (*p* < 0.05). Only variables with significant changes were selected as potential biomarkers and subjected to identification of their molecular formulas. All biomarkers were tentatively identified with the accurate mass charge ratio by the online METLIN database (http://www.metlin.scipps.edu/) (20 ppm was set as the accepted mass error). For each mass ion, several candidates were provided by the above-mentioned databases. Candidates were subjected to further MS/MS experiments (data not shown), and target molecules were validated by the characteristics of their fragment information. Then, identities were tentatively concluded based on online databases [including the METLIN database and the Massbank database (http://www.massbank.jp/)] and the literature. Finally, to identify the metabolic pathways disturbed by the administration of ANIT and rhubarb, pathway analysis of the identified potential biomarkers was performed with MetaboAnalyst 3.0 (Xia et al., 2015) (http://www.MetaboAnalyst.ca/) based on the pathway library of *Rattus norvegicus* (rat).

### Statistical analysis

The data were analyzed with the SPSS software program (version 22.0, Chicago, IL, USA). One-way analysis of variance (ANOVA) with the post hoc test followed by Student's *t*-test (the Mann–Whitney *U*-test was used when the *t*-test was not suitable) was used for the evaluation of significant differences of the results (Zhang et al., 2015; Zhao et al., 2015). The differences were considered to be statistically significant when *p* < 0.05 and highly significant when *p* < 0.01. FDR correction was not used during the univariate analysis of the metabolomics analysis because the metabolites with small *p*-values had been examined by building the classification model (Bender and Lange, 2001).

## Results

### Multicomponent quantification, fingerprint analysis, and component identification of rhubarb extract

First, the aloe-emodin, rhein, emodin, chrysophanol, and physcion contents were determined according to the Chinese Pharmacopoeia (CP) (National Pharmacopoeia Committee, 2010). The results showed that the aloe-emodin, rhein, emodin, chrysophanol and physcion contents were 0.41, 0.84, 0.36, 0.43, and 0.1%, respectively. The total content of the five anthraquinone compounds was 2.14%, which was in accord with the quality standard of the (National Pharmacopoeia Committee, 2010). Figure S1 shows the representative HPLC chromatograms of the rhubarb water extracts (Figure S1A) and the mixed standards (Figure S1B).

To ensure the quality of the rhubarb, the chemical fingerprint of the rhubarb was established. The same batches of rhubarb used for the animal studies were used for the fingerprint analysis. Ten samples were selected by random sampling; the samples were extracted twice for 20 min each time. Then, the sample solution was prepared and injected into the HPLC system. The analysis process and results were depicted in the Supplementary Materials. The results showed that the similarity values of all 10 samples were higher than 0.95, thereby proving the stability of the sample. These fingerprints can be used for future reference.

The chemical information of the rhubarb extract was established by UHPLC-MS in the ESI+ and ESI− modes, and 37 components of rhubarb were tentatively identified. The detailed content determination and UHPLC-MS identification of the components in rhubarb were depicted in the Supplementary Materials.

### Rhubarb showed an obvious therapeutic effect on rats with cholestasis

**Figure 2:**
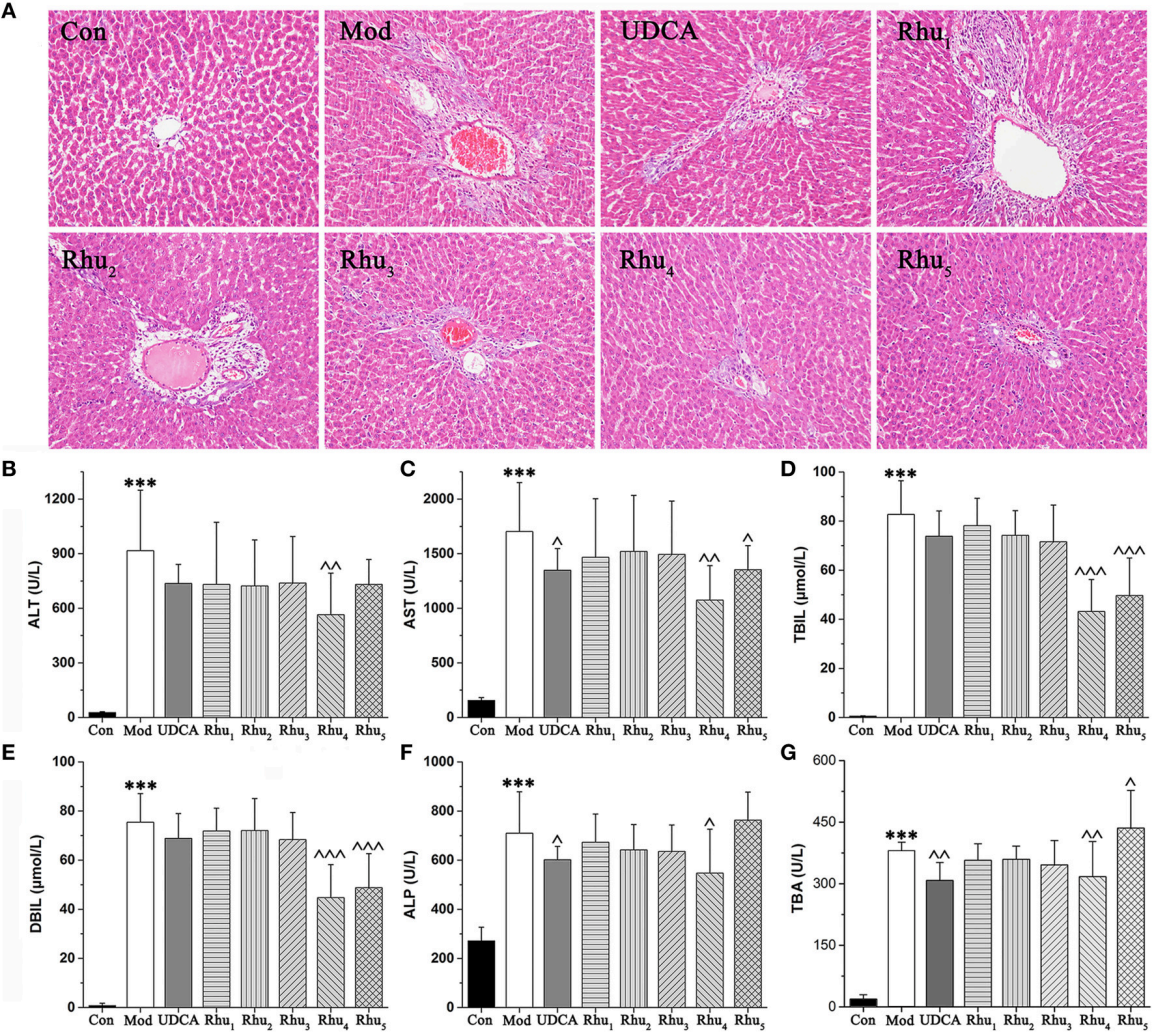
**(A)** Typical histopathological section photographs of rat liver specimens for H&E analysis (200 × magnification). **(B–G)** Shown are the serum ALT, AST, TBIL, DBIL, ALP and TBA levels, respectively. ALT, alanine aminotransferase; AST, aspartate aminotransferase; TBIL, serum total bilirubin; DBIL, serum direct bilirubin; ALP, alkaline phosphatase; TBA, total bile acid. ANOVA with the post hoc test was used to calculate the significance of the differences, ^***^ represents *p* < 0.01 compared with the control group, ^, ^^, and ^^^ represent *p* < 0.05, *p* < 0.01 and *p* < 0.001 compared with the model group, respectively.

The serum biochemistry results are presented in Table S4 and Figures 2B–G. Serum ALT and AST are well-recognized markers of various liver damages. As shown in Figures 2B,C, rats given ANIT displayed a remarkable increase in ALT and AST, which indicated that severe liver damage occurred after ANIT administration. The ALT and AST levels were significantly reduced when the rats were treated with the 6.6 and 21.0 g/kg doses of rhubarb, respectively. TBIL, DBIL, ALP and TBA, which are classical indicators for cholestatic liver injuries, were remarkably elevated in the ANIT-treated rats (Figures 2D–G), which indicated that severe cholestasis had occurred. UDCA at a 60 mg/kg dose efficiently decreased the serum levels of ALP but had a mild effect on decreasing ALT, AST, TBIL, DBIL, and TBA; thus, UDCA had a moderate treatment effect on ANIT-induced severe cholestasis. The TBIL, DBIL ALP, and TBA levels were significantly decreased in rats treated with rhubarb at a dose of 6.60 g/kg (Rhu_4_) compared with the model group. The efficacy of rhubarb at the 6.60 g/kg dose on ANIT-induced severe cholestasis was better than that of UDCA and the other rhubarb doses.

Liver tissues were examined by microscopy to provide visual evidence of the protective efficacy of rhubarb on cholestasis. As shown in Figure 2A, the liver sections of normal rats (Con) showed normal hepatocyte structures. The specimens in the model group (Mod) displayed acute neutrophil infiltration, sinusoid congestion, severe demolition, or loss of the interlobular ducts, and hepatic necrosis. The concurrent administration of UDCA and 6.6 and 22.0 g/kg of rhubarb resulted in a milder degree of bile duct epithelial damage and hepatocyte hydropic degeneration with less neutrophil infiltration, which was similar to the normal group. The specimens treated with 0.21, 0.66, and 2.10 g/kg of rhubarb displayed a slightly reduced severity of inflammatory cell infiltration and other ANIT-induced histological damages. The liver damage in the specimens treated with 0.21, 0.66, and 2.10 g/kg of rhubarb demonstrated almost no attenuation of portal tract edema, cholangitis and bile duct epithelial damage. Notably, 21.0 g/kg of rhubarb also had an obvious treatment effect in terms of some biochemical indices and histological sections. However, the efficacy of rhubarb at the 21.0 g/kg dose was inferior to rhubarb at the 6.60 g/kg dose, and the excessive dose of rhubarb was likely to generate side effects. Moreover, the TBA level was significantly elevated after administration of 21.0 g/kg of rhubarb.

Taken together, our results demonstrated that the model of cholestasis was successfully established. Rhubarb at the 6.60 g/kg dose showed a significant therapeutic effect on severe cholestasis. The therapeutic effect observed for the Rhu_4_ group (rhubarb at the 6.60 g/kg dose) was much better than the effect observed for the other groups.

### Multivariate statistical analysis of the metabolomics data

The UHPLC system provides a rapid, effective, and convenient method to analyze the chemical constituent's variance between different samples of the rats. The base peak intensity chromatograms (BPC) of samples from the control, model, and Rhu_4_ groups in positive and negative ion mode are presented in Figures S4, S5, respectively. Visual inspection of these spectra revealed differences in BPC profiles among the control, model and Rhu_4_ groups, indicating that the metabolite levels were perturbed by ANIT and rhubarb administration.

**Figure 3:**
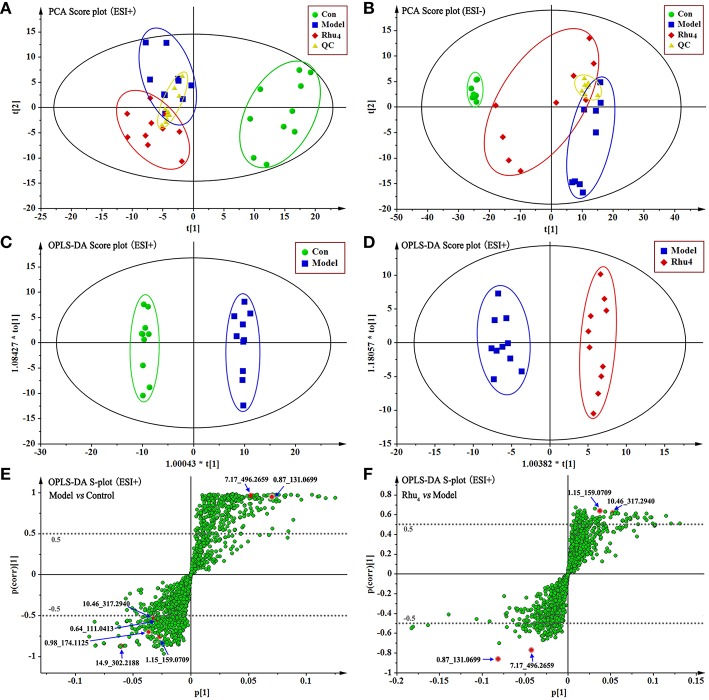
**The score plots of Con, Model, Rhu_4_, and QC from PCA in the ESI+ mode (A) and ESI− mode (B) for PC1 vs. PC2**. QC indicates the quality control group. OPLS-DA analysis of the data derived from the ESI+ mode. OPLS-DA score plots for the pair-wise comparisons between the Con and Model **(C)** and the Model and Rhu_4_ **(D)**. S-plot of the OPLS-DA model for the Con and Model **(E)** and the Model and Rhu_4_ **(F)**, the axes that are plotted in the S-plot from the predictive component are p1 vs. p(corr)1, representing the magnitude (modeled covariation) and reliability (modeled correlation) respectively. The points in red indicate the identified biomarkers.

As therapeutic effect of rhubarb at 6.60 g/kg on the cholestasis is the best. The normal, model, Rhu_4_ groups were specifically selected to get an explicit classification. At first, PCA was used as an unsupervised statistical method to study the metabolic differences between control, model and Rhu_4_ groups. The score plots of PCA analysis derived from data of ESI+ mode and ESI− mode are shown in Figures 3A,B, respectively. As shown in Figure 3, the QC samples clustered closely in both PCA score plots, demonstrating the stability of the LC/MS system throughout the whole analysis. Besides, an obvious separation trend can be observed between the Con, Mod and Rhu_4_ groups in both PCA model, indicating there was a considerable metabolite difference between the three groups.

OPLS-DA was used to investigate deep differences between control group and model group, model group and Rhu_4_ group respectively to find potential biomarkers for discriminating cholestasis and drug types. Figures 3C–F displays the result of OPLS-DA model derived from data of ESI+ analysis (M2, M3). Figures 3C,E displays the result of OPLS-DA model (M2) using the data from the control and model groups. As shown in Figure 3, the score plot (Figure 3C) showed good fitness of model. The model group can be separated from control group very clearly. The model demonstrated good predictive ability with a R^2^Y (cum) of 0.995 and Q^2^(cum) of 0.949. Similarly, the OPLS-DA model (M3) was constructed based on the model and Rhu_4_ data (Figures 3D,F). The model group can be separated from Rhu_4_ group clearly. The R^2^Y (cum) and Q^2^Y (cum) were 0.974 and 0.708 respectively. The data analysis of ESI− mode was also conducted and are shown in S5. All the parameters of PCA and OPLS-DA model are listed in Table S5.

### The exploration, selection, and tentative identification of potential biomarkers

The S-plot (Wiklund et al., 2008) and variable importance for projection (VIP) values of the OPLS-DA model were used to select the variables responsible for group separation. Variables with a VIP value > 1 were pre-selected as potential biomarkers. To decrease the risk of false positives in the selection of potential biomarkers, variables with |p(corr)| ≥ 0.5 were selected as the variables that were most correlated with the OPLS-DA discriminant scores. Figures 3E,F show the S-plots of models M2 and M3, respectively. The same process was performed for the data from the ESI− analysis; the OPLS-DA models M5 and M6 were established and the score plots and S-plots of M5 and M6 were shown in Figure S6. The M5 and M6 parameters depicted in Table S5 illustrate the good fitness of the model. By applying this analysis process, the variables responsible for group separation were selected as potential biomarker candidates.

Next, metabolites that differed significantly (*p* < 0.05) in the model group compared with the control group as well as when those in the Rhu_4_ group compared with the model group were selected as candidate biomarkers. The criteria were restricted to features with an average normalized intensity difference of 1.5-fold. Finally, the metabolites in the ESI+ and ESI− mode analyses were combined and subjected to further identification of their molecular formulas. All biomarkers were tentatively identified with the accurate mass charge ratio by the online METLIN database (http://www.metlin.scipps.edu/). To determine the potential structures of the ions, targeted MS/MS analysis was applied to identify the metabolites. The MS/MS spectra obtained at the 10, 20, and 40 eV collision energies were implemented to obtain the fragmentation patterns of these potential biomarkers.

**Figure 4:**
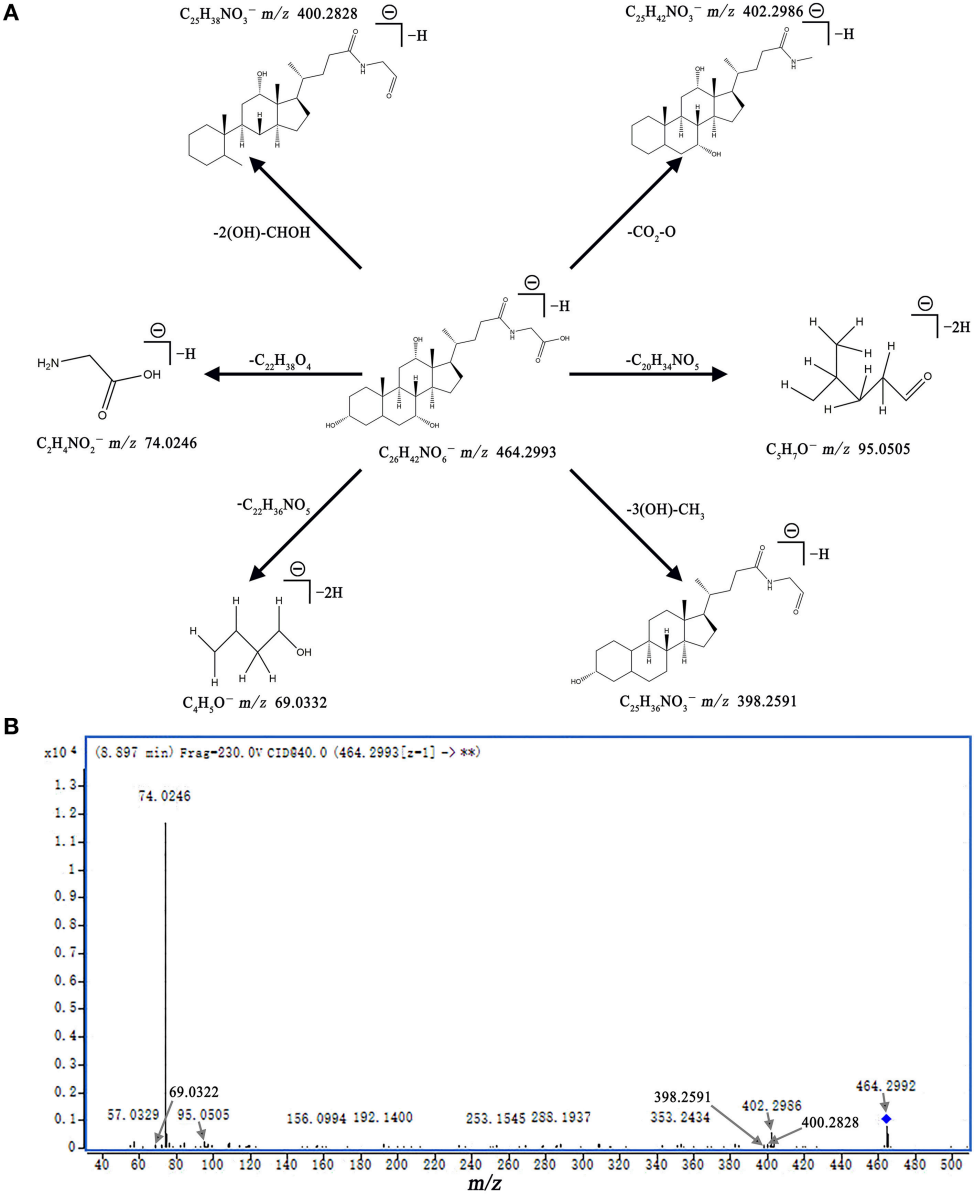
**Mass spectra and mass fragment information for glycocholic acid**. The proposed MS fragmentation mechanism **(A)** and product ion spectrum of glycocholic acid in ESI− mode **(B)**.

Metabolite identification was conducted with high resolution MS and MS/MS fragments as well as database analyses. To illustrate the identification of metabolites, we used the ion at 8.10_465.3071 with a retention time of 8.10 min and a molecular weight of 465.3071 (neutral ion) as an example. This ion may contain an odd number of nitrogen atoms because its precise molecular weight is 465.3071; its molecular formula was speculated to be C_26_H_43_NO_6_ based on the analysis of its elemental composition and fractional isotope abundance. In the negative ion spectrum, the main fragment ions analyzed via the MS/MS screening were observed at m/z 402.2986, 95.0505, 74.0246, and 69.0332, which could be the [M - H]^−^ of lost -CO_3_, -C_20_H_34_NO_5_, -C_20_H_34_NO_5_, and -C_22_H_36_NO_5_, respectively. Finally, we used the online METLIN database to define its structure and tentatively identified the metabolite as glycocholic acid. Its mass spectrum (Figure 4B) and proposed fragmentation pathway are displayed in Figure 4A. Using the protocol described above, a total of 13 potential biomarkers, including 7 metabolites from the ESI+ analysis and six metabolites from the ESI− analysis, were identified and listed in Tables 1, 2.

**Table 1:** **Differential identified metabolites for discrimination among control, model, and Rhu_4_ groups**.

**tR (min)**	**Metabolite**	**Mod vs. Con**			**Rhu**_**4**_ **vs. Mod**		
		**VIP**	**Fold**	***p*^a^**	**VIP**	**Fold**	***p*^a^**
**DATA FROM THE ESI+ MODE**							
0.64	Cytosine	1.23	0.29	0.009	<1	2.02	0.222
0.87	Creatine	2.90	3.46	<0.001	3.35	0.53	<0.001
0.98	Arginine	1.58	0.25	0.003	<1	1.77	0.440
1.15	Indoleacetaldehyde	1.15	0.45	<0.001	1.51	2.13	0.011
7.17	Leukotriene D4	2.16	55.40	<0.001	1.77	0.64	<0.001
10.46	Phytosphingosine	1.32	0.40	0.004	1.98	2.34	0.024
14.9	5(S)-HETE lactone	2.54	0.37	<0.001	1.07	0.73	0.064
**DATA FROM THE ESI− MODE**							
0.51	Sulfuric acid	<1	0.93	0.095	2.93	0.62	0.003
0.86	Taurine	7.74	0.16	<0.001	10.38	3.68	<0.001
7.63	Taurochenodeoxycholate	2.12	125.58	<0.001	2.84	0.34	0.001
7.81	Taurocholic acid	8.91	1317.39	0.001	11.49	0.39	0.009
8.10	Glycocholic Acid	1.59	39.64	<0.001	2.59	0.19	<0.001
12.97	15(S)-HETE	2.48	0.23	<0.001	2.29	1.85	<0.001

a The significant differences were generated from the Student's t-test or Mann–Whitney U-test when the Student's t-test was not suitable.

**Table 2:** **The identification of potential biomarkers based on UHPLC-MS/MS**.

**tR (min)**	**Identified compound**	**Mass (Neutral)**	**Error (ppm)**	**Formulate**	**MS/MS fragment ions (*m/z*)**
**DATA FROM THE ESI+ MODE**					
0.64	Cytosine	111.0413	17.67	C_4_H_5_N_3_O	112.3924 [M+H] ^+^ 95.0709 [M+H-NH_3_] ^+^ 67.0455 [M+H-CH_3_NO] ^+^ 52.0229 [M+H-CH_4_N_2_O] ^+^
0.87	Creatine	131.0699	−3.23	C_4_H_9_N_3_O_2_	132.0757 [M+H] ^+^ 90.0543 [M+H-CH_2_N_2_] ^+^ 58.0643 [M+H-C_2_H_4_NO_2_] ^+^
0.98	Arginine	174.1125	−4.73	C_6_H_14_N_4_O_2_	175.1193 [M+H] ^+^ 116.0704 [M+H-CH_5_N_3_]^+^
1.15	Indoleacetaldehyde	159.0709	−15.63	C_10_H_9_N_*O*_	160.0704 [M+H] ^+^ 132.0772 [M+H-CO] ^+^ 117.0539 [M+H-CO-CH_3_] ^+^
7.17	Leukotriene D4	496.2659	−10.46	C_25_H_40_N_2_O_6_S	497.2737 [M+H] ^+^
10.46	Phytosphingosine	317.2940	−3.17	C_18_H_39_NO_3_	318.2994 [M+H] ^+^, 300.2929 [M+H-H_2_O] ^+^, 256.2642 [M+H-C2H_6_O_2_] ^+^
14.9	5(S)-HETE lactone	302.2188	19.12	C_20_H_30_O_2_	303.2266 [M+H] ^+^ 105.0698 [M+H-C_12_H_22_O_2_] ^+^, 91.0543 [M+H-C_13_H_24_O_2_] ^+^ 81.0697 [M+H-C_14_H_22_O_2_] ^+^
**DATA FROM THE ESI− MODE**					
0.51	Sulfuric acid	97.9675	−1.24	H_2_SO_4_	96.961 [M-H] ^−^, 79.9579 [M-H-OH] ^−^
0.86	Taurine	125.0127	15.71	C_2_H_7_NO_3_S	124.0049 [M-H] ^−^ 79.9570 [M-H-C_2_H_6_N] ^−^
7.63	Taurochenodeoxycholate	499.2883	16.94	C_26_H_45_NO_6_S	498.2805 [M-H] ^−^ 124.0073 [M-H-C_24_H_43_O_3_] ^−^ 106.9818 [M-H-C_24_H_41_NO_3_] ^−^ 79.9575 [M-H-C_26_H_44_NO_3_] ^−^
7.81	Taurocholic acid	515.2911	1.11	C_26_H_45_NO_7_S	514.2883 [M-H] ^−^ 124.0070 [M-H-C_24_H_37_O_4_] ^−^
8.10	Glycocholic Acid	465.3071	4.16	C_26_H_43_NO_6_	464.2993 [M-H] ^−^ 402.2986 [M-H-CO_3_] ^−^ 95.0505 [M-H-C_20_H_34_NO_5_] ^−^ 74.0246 [M-H-C_22_H_38_O_4_] ^−^ 69.0332 [M-H-C_22_H_36_NO_5_] ^−^
12.97	15(S)-HETE	320.2315	11.38	C_20_H_32_O_3_	319.2256 [M-H] ^−^ 301.8645 [M-H-H_2_O] ^−^ 257.2287 [M-H-CH_2_O_3_] ^−^ 219.5090 [M-H-C_6_H_12_O] ^−^ 59.0145 [M-H-C_18_H_28_O] ^−^

### Alterations in potential biomarkers induced by different doses of rhubarb

In the evaluation of the control group and model group that sought biomarkers of ANIT-induced cholestasis (Table 1), 12 metabolites (except sulfuric acid) were significantly (*p* < 0.05) altered. The significantly altered metabolites from rats with cholestasis induced explicit metabolic changes compared with the healthy controls. Notably, treatment of cholestatic rats with 6.60 g/kg of rhubarb also induced dramatic metabolic perturbations in 11 metabolites. Through comparison, we found that 9 of the 13 biomarkers (creatine, indoleacetaldehyde, leukotriene D4, phytosphingosine, taurine, taurocholic acid, glycocholic acid, taurochenodeoxycholate, and 15(S)-HETE) were simultaneously altered during ANIT and rhubarb treatment and that the nine altered metabolites in cholestatic rats were reversed after rhubarb treatment. The results suggest that the metabolic pathways for cholestasis and rhubarb treatment are quite similar.

The results of the *in vivo* experiments demonstrated that rhubarb at a dose of 6.60 g/kg showed significant therapeutic effects on the rat model of cholestasis. The other rhubarb treatment groups also exhibited a certain efficacy against cholestasis. However, no obvious histological and biochemical changes were detected in the rats compared with the model group after treatment with a low dose of rhubarb (0.21, 0.66, and 2.10 g/kg). To obtain a deeper insight into the metabolic differences caused by the different doses of rhubarb, the changes in the above-mentioned nine potential biomarkers were further analyzed.

**Figure 5:**
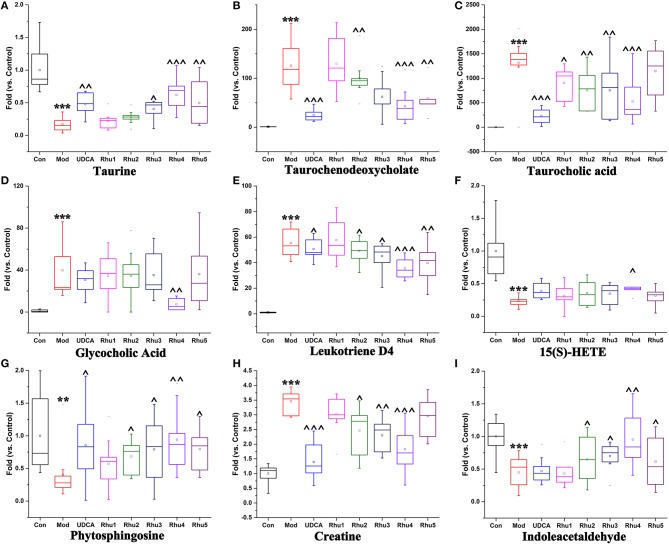
**Variations in the trends of the metabolites that are biomarkers of both cholestasis and rhubarb treatment**. **(A–I)** Shown are the variations in the trends of taurine, taurochenodeoxycholate, taurocholic acid, glycocholic acid, leukotriene D4, 15(S)-HETE, phytosphingosine, creatine and indoleacetaldehyde, respectively. ^**^ and ^***^ represent *p* < 0.01 and 0.001 compared with the control group, respectively. ^, ^^, and ^^^ represent *p* < 0.05, 0.01 and 0.001 compared with the model group, respectively.

As shown in Figures 5A–I, the taurine, indoleacetaldehyde and phytosphingosine levels were significantly elevated in the Rhu_4_ group compared with the model group; with the decrease in the rhubarb dose, the levels of the three biomarkers were gradually reduced with the reduced level of rhubarb. In contrast, the taurochenodeoxycholate, leukotriene D4 and creatine levels were significantly reduced in the Rhu_4_ group compared with the model group, and the levels of the three biomarkers were increased with the reduction in the rhubarb dose. In summary, the above-mentioned six metabolites were altered by rhubarb in a dose-dependent manner. Conversely, taurocholic acid, glycocholic acid and 15(S)-HETE did not show obvious dose-dependent changes in rats administered different doses of rhubarb.

### PCA analysis and the dose-response curves based on biomarker variations

**Figure 6:**
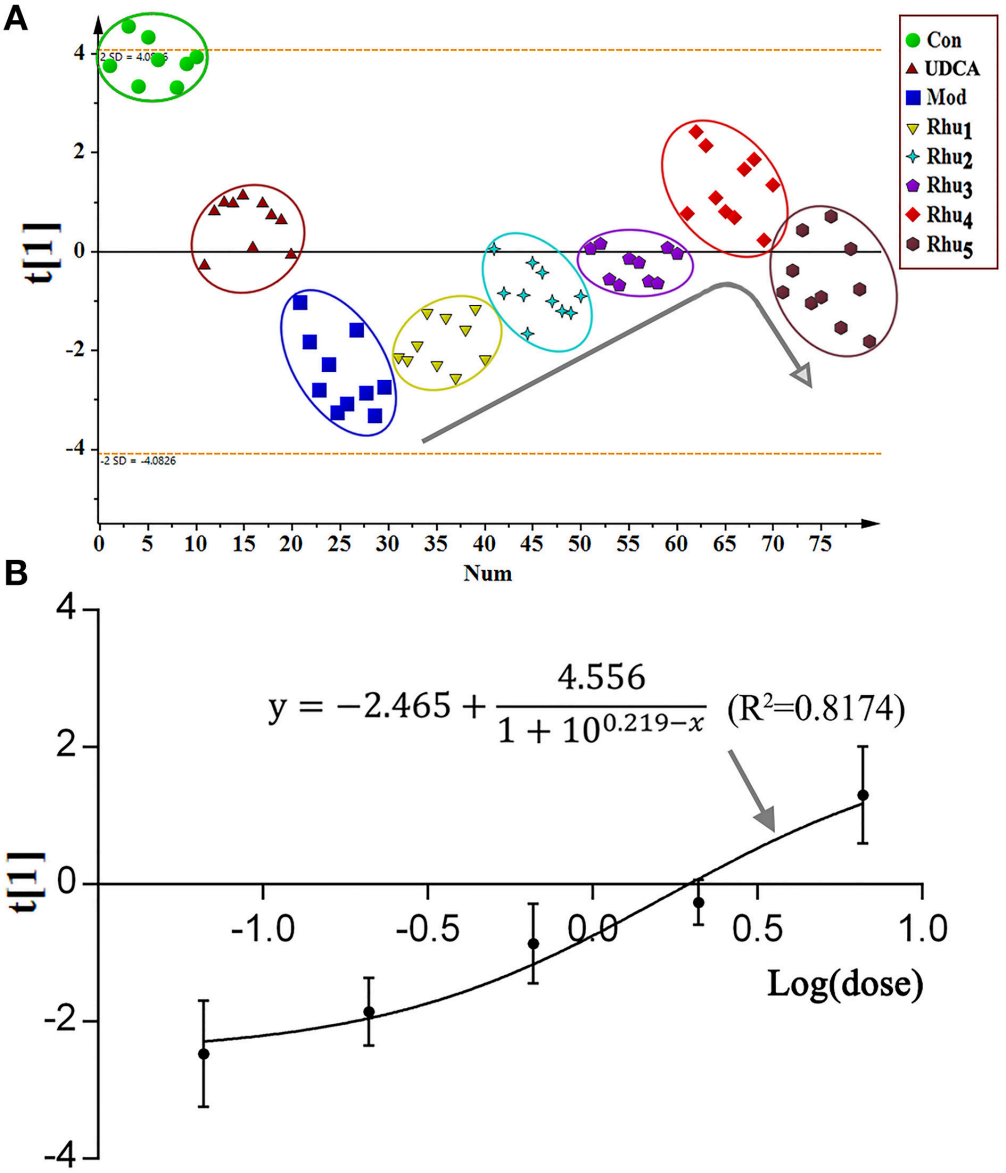
**(A)** The score plot of the PCA model. The normalized peak areas of the nine significantly altered potential biomarkers were used as the corresponding variables. All groups were highlighted by a surrounding circle in a corresponding color. The arrow in the figure shows the metabolic changes with the increasing dose of rhubarb. **(B)** The dose-response curve is provided for the effect indicator (scores of the first principal component) and rhubarb at different doses.

To evaluate the comprehensive trend and regularity of the alterations in metabolites after rhubarb treatment, the PCA model was established using the normalized peak areas of the nine important metabolites as variables. Figure 6A displays the score plot of the PCA model with the first principal component. The eight studied groups can be well-separated. The control group was separated from the model group with most of the metabolites altered (Figures 5A–I), and the positive control group showed a similar trend of separation. For the five rhubarb doses groups, rhubarb at the 0.21 g/kg dose exhibited a slight difference compared to the model group. With increased doses of rhubarb, the metabolome differences compared with the model group gradually increased. The Rhu_4_ group showed the shortest distance to the control group on the t[1]-axis, which indicated its potent therapeutic effect.

As shown in the PCA score plot, a clear dose-dependent response was observed with the doses of 0, 0.21, 0.66, 2.10, and 6.60 g/kg. The dose-response curve was established using the principal component score extracted from the PCA model as an effect indicator. The detailed modeling process of the dose-response curve was depicted in the Supplementary Materials. Figure 6B shows the fitting of the dose-response curve (R^2^ = 0.8174). As a result, the EC_20_, EC_50_, and EC_80_ values were 0.42, 3.26 and 6.61 g/kg, respectively. The 0.42–6.61 g/kg dose, which corresponded to 4.00–62.95 g in the clinic, was in the ED_20_–ED_80_ range for the treatment of cholestasis.

### Rhubarb regulates metabolic pathways with differences compared to UDCA

MetaboAnalyst, a free web-based tool that combines the topology with a powerful pathway enrichment analysis; this tool was used to perform the pathway analysis. The changes in the potential biomarkers (Table S6) suggested that nine pathways were affected by cholestasis and rhubarb treatment. As shown in Figure S7 and Table S6, the most affected pathways among the nine disturbed pathways were primary bile acid biosynthesis, taurine and hypotaurine metabolism, arachidonic acid metabolism, and arginine and proline metabolism. Figure 6 displays the schematic diagram of the disturbed metabolic pathways related to ANIT and rhubarb treatment.

**Figure 7:**
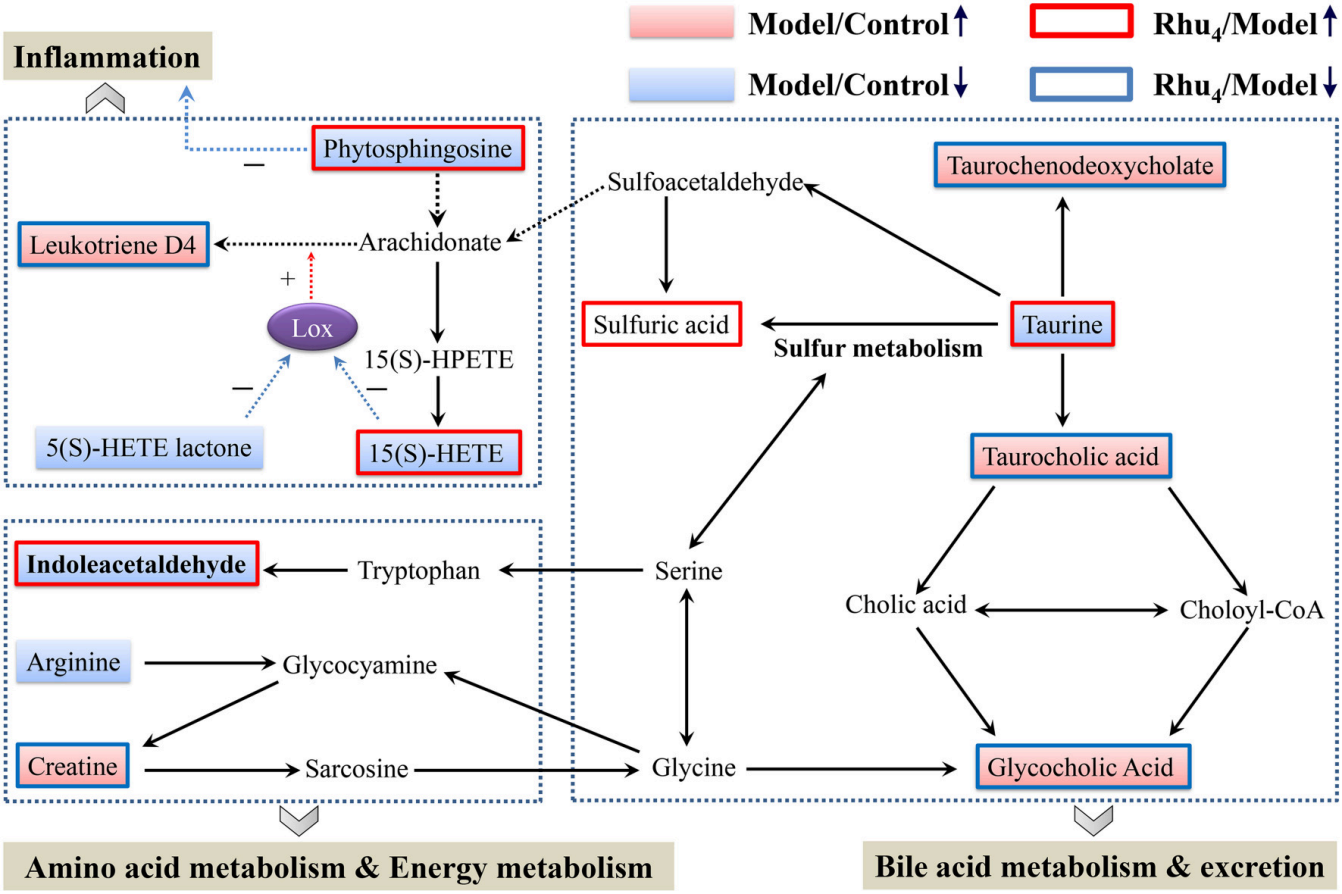
**Schematic diagram of the disturbed metabolic pathway related to ANIT and rhubarb treatment**. The pink and light blue boxes indicate metabolites significantly higher and lower in the model group than in the control group, respectively. The boxes bordered in red and blue represent metabolites that are significantly higher and lower in the Rhu_4_ group than in control group, respectively. Lox in the figure stands for lipoxygenase.

Further analysis of the disturbed biomarkers and pathways revealed that bile acid metabolism and excretion, inflammation and amino acid, metabolism and energy metabolism were generally regulated by rhubarb treatment (Figure 7). Notably, UDCA also had a considerable impact on the above-mentioned pathways. By analyzing the trends in the variation of the biomarkers, we could see that UDCA at a 60 mg/kg dose regulated bile acid metabolism and excretion more evidently than rhubarb; moreover, the taurine, taurochenodeoxycholate and taurocholic acid levels were more significantly affected by UDCA than rhubarb (Figures 5A–D). In contrast, rhubarb showed a better effect on the regulation of pathways related to inflammation and amino acid metabolism and energy metabolism. Compared with UDCA, the leukotriene D4, 15(S)-HETE, phytosphingosine, and indoleacetaldehyde levels were more significantly perturbed by rhubarb (Figures 5E–I).

## Discussion

Cholestasis is characterized by the intrahepatic accumulation of potentially toxic bile acids as a consequence of hepatocellular dysfunction or the obstruction of bile ducts (Modica et al., 2012). The complications and severity of the pathological implications make cholestasis an intractable disease (Hegade and Jones, 2015). In the present study, UDCA at a 60 mg/kg dose showed an anti-cholestasis effect on the ANIT-induced cholestasis model to a certain degree. Rhubarb at a 6.6 g/kg dose (measured as the quantity of crude material) displayed a remarkable therapeutic effect, and rhubarb showed an obvious dose-dependent effect on cholestasis. With the increase in the dose, the anti-cholestasis effect gradually increased. However, a larger dose did not mean better efficacy; the 6.60 g/kg dose might be the optimal dose for rats because the dose higher than 6.60 g/kg used in this study (21.0 g/kg) exhibited a drop in efficacy and might lead to side effects. Furthermore, the established dose-response curve showed that 0.42–6.61 g/kg (corresponding to 4.00–62.95 g in the clinic) was in the EC_20_–EC_80_ range for cholestasis treatment. However, the regular dose of rhubarb in Chinese Pharmacopoeia was 3–15 g, which could not achieve a very good therapeutic effect on cholestasis. Our previous study demonstrated that a large dose of rhubarb over a short period of time did not cause obvious toxicity (Wang et al., 2011a), thus a broader dosage range was recommended for the Chinese Pharmacopoeia.

In the evaluation of control group and model group for seeking the biomarker of ANIT-induced cholestasis (Table 1), 12 metabolites (except sulfuric acid) were significantly (*p* < 0.05) alerted. The significantly alerted metabolites illustrating rats with cholestasis induced explicit metabolic change compared with the healthy controls. The changed concentrations of potential biomarkers suggested that the disturbed pathways in cholestasis rats, such as primary bile acid biosynthesis, taurine, and hypotaurine metabolism, arachidonic acid metabolism, and arginine and proline metabolism, were most affected by the administration of ANIT. As noted in two previous reports (Chen et al., 2016; Ma et al., 2016), primary bile acid biosynthesis, taurine and hypotaurine metabolism and the pathways related to inflammation and amino acid metabolism were also disturbed in cholestasis rats.

Because bile acids are closely related to the occurrence and development of cholestasis, special attention has been paid to the bile acid level related to the treatment of cholestasis (Thomas et al., 2008). To evaluate the protective effect of rhubarb against ANIT-induced liver injury, a previous study based on the targeted metabolic profiles of 8 bile acids partially clarified the mechanisms of cholestasis and the therapeutic effect of rhubarb (Yang et al., 2012). In this study, the importance of bile acids was also observed. Five identified biomarkers (taurine, taurochenodeoxycholate, taurocholic acid, glycocholic acid, and sulfuric acid) were involved in primary bile acid biosynthesis or taurine and hypotaurine metabolism (Figure 7).

Taurochenodeoxycholate, taurocholic acid and glycocholic acid are common primary bile acids synthetized from cholesterol and conjugated with taurine or glycine in hepatocytes. The enhancement of taurochenodeoxycholate, taurocholic acid, and glycocholic acid together with the reduced level of taurine are commonly present in cholestasis patients (Trottier et al., 2012; Woolbright et al., 2015). Further analysis showed that the higher level of taurine together with the lower level of taurochenodeoxycholate, taurocholic acid, and glycocholic acid in the Rhu_4_ group compared with the model group might suggest that the therapeutic mechanism of action of rhubarb occurs via bile acid synthesis and secretion. Interestingly, the taurine, taurochenodeoxycholate, and taurocholic acid levels exhibited an obvious dose-dependent change after rhubarb treatment, indicating that primary bile acid biosynthesis, and taurine and hypotaurine metabolism were affected in a dose-dependent manner.

In addition to the biomarkers associated with bile acid metabolism and excretion, biomarkers related to inflammation were also identified by the untargeted metabolomics approach. The observations of the significantly reduced levels of 15(S)-HETE, phytospingosine, and 5(S)-HETE lactone together with the significantly elevated level of leukotriene D4 (LTD_4_) in the model group indicated the dysfunction of arachidonic acid metabolism. 15(S)-HETE is a hydroxyeicosatetraenoic acid that can inhibit leukotriene B4 (LTB_4_) formation and LTB_4_-induced erythema and edema (Samuelsson et al., 1987). Studies have shown that 15(S)-HETE and 5(S)-HETE lactone can inhibit lipoxygenase (Lox) activity and further inhibit the production of LTD_4_ (Sekiya and Okuda, 1982; Kerdesky et al., 1987; Dwyer et al., 2004) (Figure 7). LTD_4_ is a potent inflammatory mediator that is associated with the pathogenesis of several inflammatory disorders (Samuelsson, 1983). In addition, we also identified phytosphingosine, which is a metabolite with anti-inflammatory activity (Pavicic et al., 2007). The elevated levels of phytospingosine and 5(S)-HETE together with the reduced level of LTD_4_ after rhubarb treatment further defined the anti-inflammatory effect of rhubarb. Moreover, the levels of phytospingosine and LTD_4_ were dose-dependent, indicating that arachidonic acid metabolism, which is closely related to the inflammatory response, was also affected by rhubarb in a dose-dependent manner.

As shown in Figure 7, amino acid metabolism was affected in ANIT-induced cholestasis. The reduced level of indoleacetaldehyde indicated that tryptophan metabolism was influenced. In addition, the increased level of creatine and reduced level of arginine indicated that a dysfunction in arginine and proline metabolism was induced by ANIT treatment. Previous studies demonstrated the protective effects of arginine on cholestatic rats (Ozsoy et al., 2011). Notably, the elevated level of creatine suggested a facilitated utilization of creatine-phosphate to replenish the energy demand; this phenomenon has been proposed to be a sign of liver injury, which was consistent with the pharmacological results in the present study (Jiang et al., 2013; Wei et al., 2015). The creatine and indoleacetaldehyde levels in response to rhubarb treatment showed an obvious dose-dependent trend, indicating that the pathways related to amino acid metabolism and energy metabolism were affected in a dose-dependent manner. Based on the dose-dependent alterations in biomarkers and metabolic pathways following treatment with different rhubarb doses, we attempted to elucidate the possible mechanism underlying the dose-response relationship and the therapeutic mechanism of action of rhubarb for the treatment of cholestasis. The results showed that the untargeted metabolomics approach used in this study might efficiently provide insights into the mechanisms. However, future metabolomic studies in human populations with cholestasis will be needed to validate the biomarkers found in the animal model. And further work should be performed to confirm the alterations in the metabolic pathways using molecular biology approaches.

## Conclusion

The results of the serum biochemistry and histopathology analyses demonstrated the conspicuous anti-cholestatic effects of rhubarb. By considering the pharmacological indicators together, we observed a clear dose-response relationship. However, a larger dose did not indicate a better therapeutic effect because the 6.60 g/kg dose in rats (which was a relatively large dose corresponding to a dose of approximately 60 g per day for a human weighing 60 kg) was the optimal dose for cholestasis treatment, and the effect of rhubarb at the 21.0 g/kg dose was inferior to that of the 6.60 g/kg dose.

An untargeted metabolomics approach based on UHPLC-MS coupled with pathway analysis was developed and successfully applied to explore the biological changes induced by different doses of rhubarb on ANIT-induced cholestasis. Based on the information extracted through the multivariate analysis, differences in the metabolic changes were detected. Altogether, 13 significantly changed metabolites, including creatine, taurine, and glycocholic acid, were identified as potential biomarkers of cholestasis or rhubarb treatment. A total of 9 of the 13 biomarkers were simultaneously altered with opposing trends in variation after ANIT and rhubarb treatment. The dose-response curve based on the PCA model of the nine important biomarkers indicated that a dose ranging from 0.42 to 6.61 g/kg (corresponding to 4.00–62.95 g in the clinic) was in the EC_20_–EC_80_ range for cholestasis treatment.

Furthermore, the pathway analysis indicated that the ANIT-induced cholestasis and rhubarb treatment were mainly responsible for alterations in bile acid metabolism and excretion, inflammation and amino acid metabolism, and energy metabolism. The levels of the identified biomarkers indicated that important pathways were perturbed in a dose-dependent manner. Notably, rhubarb showed a poorer effect on bile acid metabolism and excretion and a better effect on the regulation of pathways related to inflammation and amino acid metabolism and energy metabolism compared with UDCA at a 60 mg/kg dose. The altered metabolites and pathways related to bile acid metabolism and excretion, inflammation and amino acid metabolism, and energy metabolism may partially clarify the therapeutic mechanism of action of rhubarb for the treatment of cholestasis.

## Author contributions

CZ, MN performed the investigation, analyzed the data and wrote the paper; JW, XD and XX designed the study and amended the paper; RL, WF, QD, GL, YM, YW, PY, YL, and LH helped in execution of research; JW, XD, XX, YZ, and all the other authors read, improved and approved the manuscript.

### Conflict of interest statement

The authors declare that the research was conducted in the absence of any commercial or financial relationships that could be construed as a potential conflict of interest. The reviewer, DR, and handling Editor, declared their shared affiliation, and the handling Editor states that the process nevertheless met the standards of a fair and objective review.
