# Supplementary material for: Untargeted Metabolomics Reveals Dose-Response Characteristics for Effect of Rhubarb in a Rat Model of Cholestasis
Source: Front Pharmacol. 2016 Mar 31;7:85. doi: 10.3389/fphar.2016.00085 (PMC4814850; doi:10.3389/fphar.2016.00085)
Supplement: Supplementary file 1 [file DataSheet1.docx]

***Supplementary materials for*：**

**Untargeted metabolomics reveals** **dose-response characteristics for** **effect of rhubarb** **in a rat model of cholestasis**

Cong-En Zhang^1, 2‡^, Ming Niu^2‡^, Rui-Yu Li^1,2^, Wu-Wen Feng^1,2^, Xiao Ma^1,2^, Qin Dong^1,2^, Zhi-Jie Ma^2,3^, Guang-Quan Li^1,2^, Ya-Kun Meng^2^, Ya Wang^2^, Ping Yin^2^, Lan-Zhi He^2^, Yu-Meng Li^2^, Peng Tan^2^, Yan-Ling Zhao^2^, Jia-Bo Wang^2*^, Xiao-Ping Dong^1*^, Xiao-He Xiao^2*^

*^1^* *College of Pharmacy, Chengdu University of Traditional Chinese Medicine, Chengdu, PR China.*

*^2^ China Military Institute of Chinese Medicine, 302 Military Hospital, Beijing, PR China.*

*^3^ Department of Pharmacy, Beijing Friendship Hospital, Capital Medical University, Beijing, PR China.*

Correspondence:

Jia-Bo Wang and Xiao-He Xiao,

302 Military Hospital, No. 100 Xisihuan Beijing 100039, China

Fax: +86 66933322; +86 66933325

E-mail: pharm_sci@126.com; pharmacy302xxh@126.com.

Xiao-Ping Dong, College of Pharmacy, Chengdu University of Traditional Chinese Medicine.

E-mail: dongxiaoping11@126.com, Tel/Fax: +86: +86 10 66933 324;

‡ These authors contributed equally to this work.

* To whom correspondence should be addressed.

# Multicomponent quantification of the rhubarb by HPLC

Analyses were performed using an Agilent 1200 HPLC system (Agilent Technologies, Santa Clara, California, USA). Chromatography was carried out at 30ºC on a Kromasill 100-5 C18 column (250mm × 4.6mm, with 5 μm particle size). The mobile phase consisted of (A) methanol and (B) 0.01% (v/v) phosphoric acid water solution. The gradient elution was as follows: 70% A from 0 to 11 min, 70-85% A from 11 to 13 min, 85% A from 13 to 22 min, 85-95% A from 22 to 24 min, at a flow rate of 1.0 mL⋅min^−1^. The signal was monitored at 254 nm.

Standards of [aloe-emodin](javascript:void(0);), [rhein](javascript:void(0);), emodin, [chrysophanol](javascript:void(0);) and [physcion](javascript:void(0);) were purchased from the Chengdu Pufei De Biotech., Ltd (Chengdu, Sichuan, China) and the purity of all these compounds were higher than 98.0%. HPLC-grade methanol was purchased from Burdick & Jackson (Ulsan, Korea.). Water was purified by a Milli-Q Plus water purification system (Millipore, USA). AR-grade phosphoric acid was obtained from [Xilong Chemical Co., Ltd.](http://xilongchemical.en.made-in-china.com/) (Beijing, China).

**Figure S1A** shows the HPLC profile of rhubarb extract. **Figure S1B** shows the chromatogram of [aloe-emodin](javascript:void(0);), [rhein](javascript:void(0);), emodin, [chrysophanol](javascript:void(0);) and [physcion](javascript:void(0);) standard mixture. As a result, the concentrations of [aloe-emodin](javascript:void(0);), [rhein](javascript:void(0);), emodin, [chrysophanol](javascript:void(0);) and [physcion](javascript:void(0);) in rhubarb were 0.41%, 0.84%, 0.36%, 0.43% and 0.1%, respectively. The total content of the five anthraquinone compounds were 2.14%, which was in accord with the quality standard of Chinese Pharmacopoeia (CP) (2010).


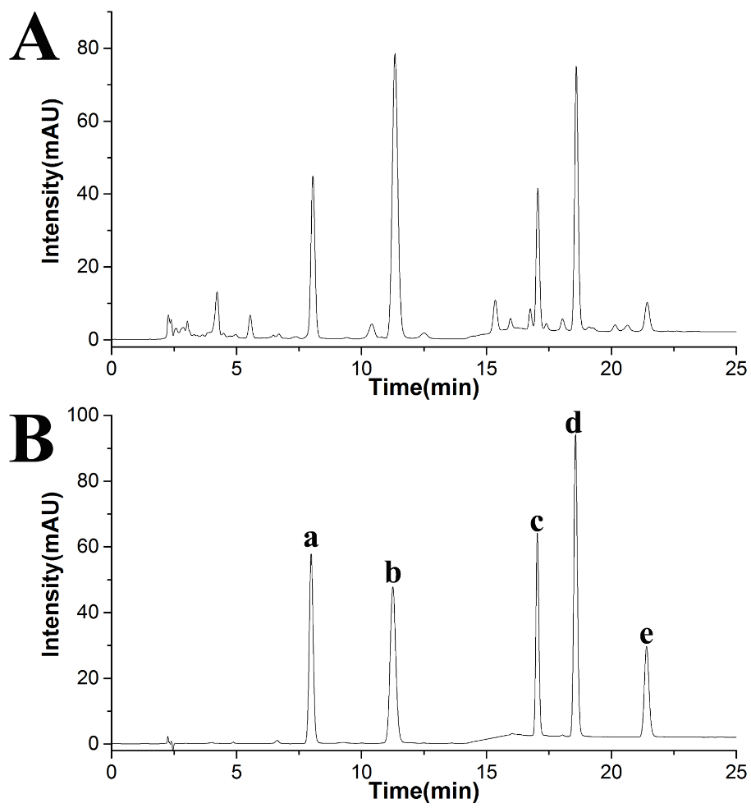


**Figure S1**. Multicomponent quantification of the rhubarb. (**A**) HPLC profile of rhubarb extract. (**B**) Chromatogram of [aloe-emodin](javascript:void(0);), [rhein](javascript:void(0);), emodin, [chrysophanol](javascript:void(0);) and [physcion](javascript:void(0);) standard mixture, peak a for [aloe-emodin](javascript:void(0);), peak b for [rhein](javascript:void(0);), peak c for [chrysophanol](javascript:void(0);), peak d for [chrysophanol](javascript:void(0);), and peak e for [physcion](javascript:void(0);), respectively.

# The HPLC finger print analysis of rhubarb

The analysis was performed using Agilent 1200 HPLC system. Chromatography was carried out at 30ºC on a Kromasill 100-5 C18 column (250mm×4.6mm, with 5 μm particle size). The mobile phases used were solvent A (methanol), solvent B (water spiked with 0.01% phosphoric acid), with gradient elution as follows: 5% – 30% A at 0 – 12.5 min, 30% – 60% A at 12.5 – 30 min, 60% – 70% A at 35–50 min, 70% –100% A at 50–60 min, 100% A at 60–65 min. The flow rate was kept at 1 mL⋅min^−1^. The signal was monitored 280 nm. The injection volume of samples was 10 μL.

Professional software "Similarity Evaluation System for Chromatographic Fingerprint of Traditional Chinese Medicine" (Version 2004A, SES software) was used for evaluating the similarities between different samples. The reference chromatogram was generated with average data.

Sample no. 1 was selected as a representative sample to validate the method for fingerprint analysis. Method precision and reproducibility were evaluated by the analysis of five injections of the sample solution and five sample solution prepared independently from sample no. 1, respectively. RSD values of the relative retention time (RRT) and the relative peak area (RPA) for some characteristic peaks, including 6 peaks (**Figure S2B**, peak no. 1, 2, 3, 4, 5, 6) were calculated. And the stability study of the sample was performed within 24h (0, 4, 8, 12, 24 h). RSDs of RRT and RPA in the precision test (n = 6) were found in the range of 0.13-0.27% and 1.70-5.11%, respectively. Reproducibility (n = 6) for both RRT (0.11 - 0.24% RSD) and RPA (1.86 - 4.55% RSD) were acceptable. Stability (RSD) was found to be ≤ 0.36% for RRT and ≤ 4.55% for RPA, indicating that the sample was stable for 24h. In total, the RSDs of RTT were less than 0.36% < 2% and the RSDs of RPA were less than 5.43% < 7%, thus the good precision, reproducibility and stability of analysis were demonstrated. **Table S1** summarizes the obtained data.

**Table S1** Injection precision, reproducibility and stability fingerprint analysis

| Peak no. | RSDs of RRT (%) (n=5) | | |  | RSDs of RPA (%) (n=5) | | |
| --- | --- | --- | --- | --- | --- | --- | --- |
|  | Precision | Reproducibility | Stability |  | Precision | Reproducibility | Stability |
| 1 | 0.27 | 0.23 | 0.15 |  | 5.11 | 3.58 | 5.43 |
| 2 | 0.12 | 0.15 | 0.25 |  | 2.15 | 2.56 | 3.01 |
| 3 | 0.19 | 0.16 | 0.32 |  | 4.32 | 1.86 | 2.06 |
| 4 | 0.13 | 0.11 | 0.22 |  | 3.53 | 4.55 | 4.29 |
| 5 | 0.17 | 0.24 | 0.25 |  | 1.70 | 2.21 | 3.45 |
| 6 | 0.18 | 0.15 | 0.36 |  | 1.77 | 1.99 | 2.53 |

The fingerprint of rhubarb was then established (**Figure S2**). **Table S2** shows the values of similarity of each sample. The closer the similarity values to 1, the more similar the chromatogram to the reference chromatogram. As is shown in **Table S2**, the similarity values of all the 10 samples was higher than 0.95, the stability of sample was proved.


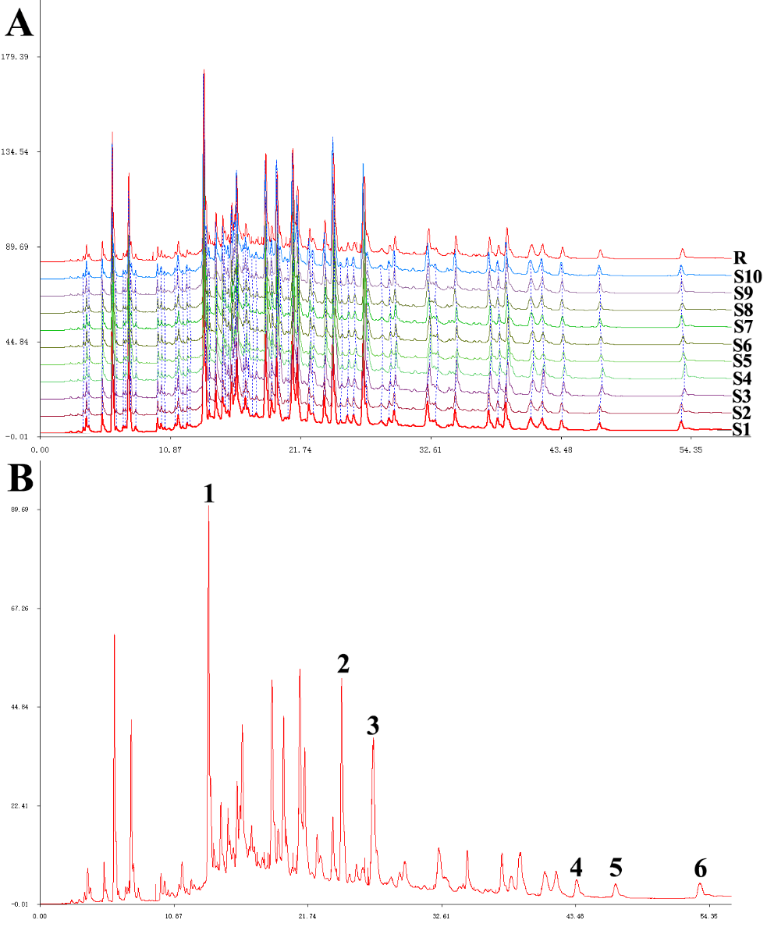


**Figure S2**. Chemical fingerprint of rhubarb. Sample nos. 1-10 (S1-S10) were used to construct fingerprint (**A**). R stands for the reference chromatogram. (**B**) Shown is the reference fingerprint, peaks no. 1-6 were used to valid the method.

**Table S2** The similarities of chromatograms of each sample.

| No. | Similarity | No. | Similarity |
| --- | --- | --- | --- |
| 1 | 0.97 | 6 | 0.98 |
| 2 | 0.97 | 7 | 0.97 |
| 3 | 0.98 | 8 | 0.98 |
| 4 | 0.98 | 9 | 0.97 |
| 5 | 0.96 | 10 | 0.97 |

# LC-QTOF/MS identification for the phytochemicals of the water extract of rhubarb

The LC–MS analysis was performed using Agilent 1290 series UHPLC system coupled to 6550 Q-TOF/MS mass spectrometer. The analysis was conducted on a ZORBAX RRHD 300 SB-C18 column (2.1×100 mm, 1.8 μm). For the ESI+ analysis, the mobile phases used were solvent A (Acetonitrile spiked with 0.1% [formic](javascript:void(0);) [acid](javascript:void(0);)), solvent B (Water spiked with 0.1% [formic](javascript:void(0);) [acid](javascript:void(0);)), with gradient elution as follows: 25% A at 0 – 15 min, 25 % – 60% A at 15 – 20 min, 60 % – 95% A at 20–25 min, 95% A at 25–30 min. The flow rate was kept at 0.3 mL/min. The column and autosampler were maintained at 30ºC and 4°C, respectively. The injection volume of reference compounds and samples was 1 μL. For the ESI- analysis, the mobile phases used were solvent A (CH_3_OH) and solvent B (H_2_O), the other analysis conditions were identical to ESI+ analysis.

A high resolution electrospray mass spectrometer was operated both in negative (ESI-) ion mode for LC-MS analysis. For the full-scan MS analysis, the spectra was recorded in the range of m/z 50-1200. The optimal conditions of analysis were as follows: the capillary voltage was 4 kV; desolvation gas flow was 11.0 L/min, the source temperature was set at 125ºC, the desolvation gas temperature was 225ºC. Calibrations were automatically conducted from m/z 50 to 1200 with a solution of sodium formate.

To reveal the identities of the principal active components in the extract of rhubarb, chemical constituents were tentatively identified based on accurate mass and referring to the related literatures. 28 common peaks of ESI+ analysis and 22 common peaks of ESI- analysis, altogether 37 compounds were tentatively identified. The mass data and compounds identified from the peaks are summarized in **Figure S3** and **Table S3**.


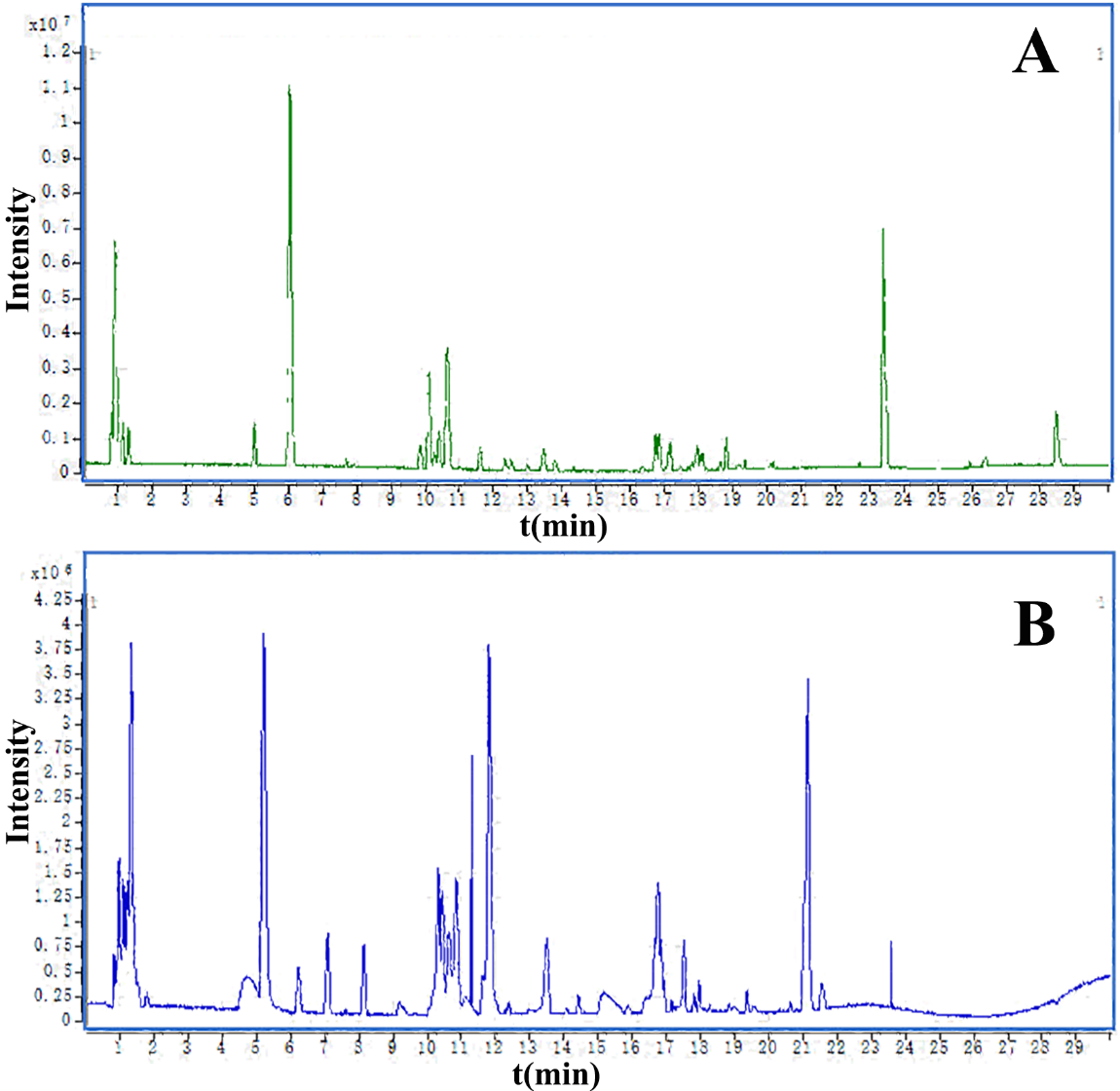


**Figure S3**. The base peak chromatogram of LC-MS analysis of rhubarb extract in ESI+ mode (A) and ESI- mode (B).

**Table S3**. Tentative identification of components contained in the extract of rhubarb

| No | Positive mode | |  | Negative mode | | M | Formulate | Identification |
| --- | --- | --- | --- | --- | --- | --- | --- | --- |
|  | tR(min) | M+H |  | tR(min) | M-H |  |  |  |
| 1 | 4.32 | 453.1397 |  | 3.23 | 451.1240 | 452.1319 | C_21_H_24_O_11_ | (+)-Catechin-5-O-glucoside |
| 2 | 6.09 | 443.0978 |  | - | - | 442.0900 | C_22_H_18_O_10_ | (-)-Epicatechin-3-O-gallate |
| 3 | 5.35 | 485.0931 |  | - | - | 484.0853 | C_20_H_20_O_14_ | 1,6-Di-O-galloyl-β-glucose |
| 4 | - | - |  | 1.11 | 331.0665 | 332.0744 | C_13_H_16_O_10_ | 1-O-Galloyl-glucose |
| 5 | - | - |  | 2.50 | 243.0504 | 244.0583 | C_10_H_12_O_7_ | 1-O-Galloyl-glycerol |
| 6 | 9.35 | 311.1131 |  | - | - | 310.1053 | C_15_H_18_O_7_ | 2-Cinnamoyl-glucose |
| 7 | 8.96 | 237.0763 |  | - | - | 236.0685 | C_12_H_12_O_5_ | 2-Methyl-5-carboxymethyl-7-hydroxychromanone |
| 8 | 11.41 | 463.1240 |  | - | - | 462.1162 | C_22_H_22_O_11_ | 2-O-Cinnamoyl-glucogallin |
| 9 | - | - |  | 6.19 | 163.0759 | 164.0837 | C_10_H_12_O_2_ | 4-(4-hydroxyphenyl)-2-butanone |
| 10 | 9.73 | 235.0970 |  | - | - | 234.0892 | C_13_H_14_O_4_ | 5-Carboxy-7-hydroxy-2-methyl-benzopyran-γ-one |
| 11 | 11.67 | 395.1342 |  | - | - | 394.1264 | C_19_H_22_O_9_ | 6-Hydroxymusizin-8-O-β-D-glucoside |
| 12 | 1.48 | 333.0822 |  | 1.34 | 331.0665 | 332.0744 | C_13_H_16_O_10_ | 6-O-Galloyl-glucose |
| 13 | 9.73 | 235.0970 |  | - | - | 234.0892 | C_13_H_14_O_4_ | 7-Hydroxy-2-(2-hydroxy)propyl-5-methyl-benzopyran-γ-one |
| 14 | - | - |  | 19.36 | 269.0450 | 270.0528 | C_15_H_10_O_5_ | Aloeemodin |
| 15 | 16.74 | 433.1135 |  | 16.69 | 431.0978 | 432.1056 | C_21_H_20_O_10_ | Aloeemodin-ω-O-β-D-glucopyranoside |
| 16 | 11.54 | 233.0814 |  | 11.3 | 231.0657 | 232.0736 | C_13_H_12_O_4_ | Cassiachromone |
| 17 | 9.09 | 443.0978 |  | 10.69 | 441.0822 | 442.0900 | C_22_H_18_O_10_ | catechin-3-O-gallate |
| 18 | 16.82 | 255.0657 |  | 16.98 | 253.0501 | 254.0579 | C_15_H_10_O_4_ | Chrysophanol |
| 19 | 11.54 | 417.1186 |  | - | - | 416.1107 | C_21_H_20_O_9_ | Chrysophanol-1-O-β-D-glucoside |
| 20 | - | - |  | 15.08 | 239.0344 | 240.0423 | C_14_H_8_O_4_ | danthron,(1,8-dihydroxyanthraquinone) |
| 21 | - | - |  | 21.06 | 269.0450 | 270.0528 | C_15_H_10_O_5_ | Emodin |
| 22 | - | - |  | 10.07 | 431.0978 | 432.1056 | C_21_H_20_O_10_ | Emodin-1-O-β-D-glucopyranoside |
| 23 | 4.71 | 291.0869 |  | 5.67 | 289.0712 | 290.0790 | C_15_H_14_O_6_ | Epicatechin |
| 24 | - | - |  | 0.96 | 169.0137 | 170.0215 | C_7_H_6_O_5_ | Gallic acid |
| 25 | 5.35 | 485.0931 |  | 3.21 | 483.0775 | 484.0853 | C_20_H_20_O_14_ | Gallic acid-3-O-(6'-O-galloyl)glucoside |
| 26 | 8.96 | 479.1553 |  | 10.32 | 477.1397 | 478.1475 | C_23_H_26_O_11_ | Lindleyin |
| 27 | 6.51 | 379.1393 |  | - | - | 378.1315 | C_19_H_22_O_8_ | Musizin-8-O-β-D-glucoside |
| 28 | 16.57 | 495.1444 |  | - | - | 494.1366 | C_30_H_22_O_7_ | Palmidin B |
| 29 | 5.74 | 327.1444 |  | 6.18 | 325.1287 | 326.1366 | C_16_H_22_O_7_ | Phenylbutanone-glucoside |
| 30 | 18.24 | 285.0763 |  | 23.40 | 283.0606 | 284.0685 | C_16_H_12_O_5_ | Physcion |
| 31 | 7.41 | 731.1612 |  | 7.58 | 729.1456 | 730.1534 | C_37_H_30_O_16_ | Procyanidin B1 3'-O-gallate |
| 32 | 8.7 | 883.1722 |  | - | - | 882.1644 | C_44_H_34_O_20_ | Procyanidin B2 3,3'-di-O-gallate |
| 33 | - | - |  | 15.11 | 283.0243 | 284.0321 | C_15_H_8_O_6_ | Rhein |
| 34 | 10.64 | 539.0978 |  | - | - | 538.0900 | C_30_H_18_O_10_ | Sennidin A |
| 35 | 10.64 | 863.2035 |  | - | - | 862.1957 | C_42_H_38_O_20_ | Sennoside B |
| 36 | 10.25 | 849.2242 |  | - | - | 848.2164 | C_42_H_40_O_19_ | Sennoside C |
| 37 | 14.63 | 409.1499 |  | 16.95 | 407.1342 | 408.1420 | C_20_H_24_O_9_ | Torachrysone-8-o-beta-d-glucoside |

# LC-MS Analysis of Metabolic Profiling

Using the optimal LC-MS, global metabolic profiling in both positive and negative ion modes were analyzed by UHPLC-MS, the base peak intensity chromatograms (BPC) of samples from the control, model, and Rhu_4_ groups in positive and negative ion mode are presented in **Figure S4** and **Figure S5**, respectively.


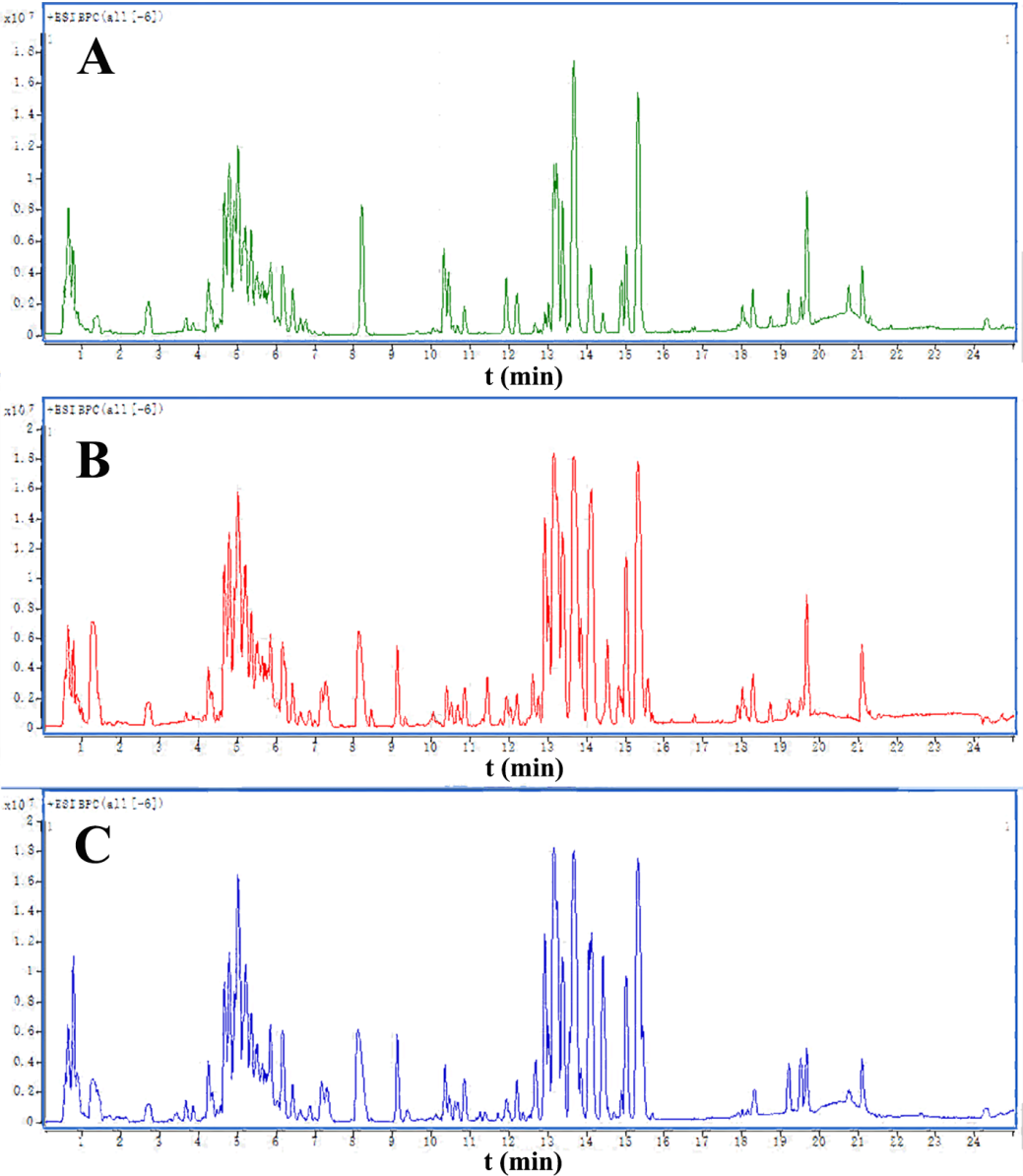


**Figure S4**. The representative BPC chromatograms of samples from Control, Model and Rhu_4_ groups analyzed on UHPLC-QTOF/MS in ESI+ mode.


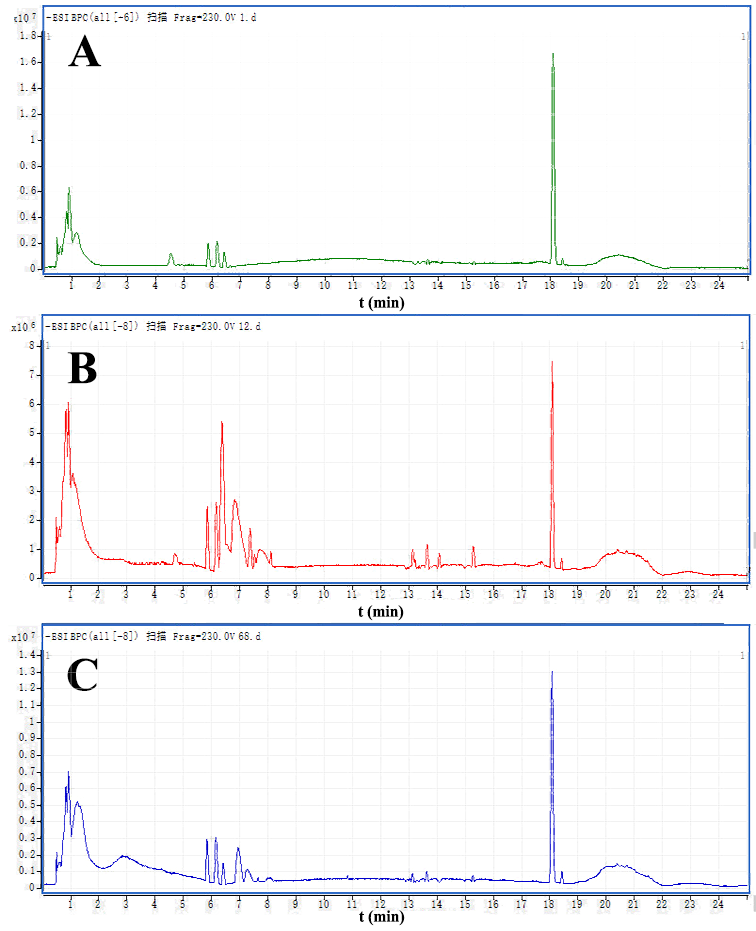


**Figure S5**. The representative BPC chromatograms of samples from Control, Model and Rhu_4_ groups analyzed on UHPLC-QTOF/MS in ESI- mode.

1. **The detailed biochemical indicators of all the group**

**Table S4** Protective effect of rhubarb against ANIT-induced cholestasis with regarded to biochemical indices

| Groups | ALT (U/L) | AST (U/L) | TBIL (μmoI/L) | DBIL (μmoI/L) | ALP (μmoI/L) | TBA (μmoI/L) |
| --- | --- | --- | --- | --- | --- | --- |
| Con | 27.10 ± 5.07 | 156.70 ± 25.45 | 0.58 ± 0.10 | 0.75 ± 0.98 | 271.50 ± 55.31 | 19.00 ± 10.43 |
| Mod | 917.10 ± 332.09^**^ | 1704.20 ± 446.97^**^ | 82.72 ± 13.68^**^ | 75.44 ± 11.66^**^ | 709.80 ± 168.62^**^ | 366.70 ± 37.68^**^ |
| UDCA | 736.50 ± 105.05 | 1348.30 ± 198.70^#^ | 73.83 ± 10.31 | 68.89 ± 10.04 | 601.90 ± 54.37^#^ | 338.00 ± 65.31 |
| Rhu_1_ | 731.50 ± 341.36 | 1467.30 ± 537.35 | 78.18 ± 11.10 | 71.85 ± 9.28 | 673.10 ± 115.08 | 357.40 ± 39.96 |
| Rhu_2_ | 723.20 ± 252.13 | 1521.00 ± 513.40 | 74.17 ± 10.11 | 72.03 ± 13.01 | 642.30 ± 102.56 | 359.60 ± 31.88 |
| Rhu_3_ | 738.90 ± 255.48 | 1494.20 ± 488.55 | 71.57 ± 14.95^#^ | 68.38 ± 11.03 | 636.10 ± 107.45 | 355.40 ± 59.78 |
| Rhu_4_ | 564.85 ± 228.39^##^ | 1074.46 ± 316.37^##^ | 43.15 ± 13.04^##^ | 44.72 ± 13.46^##^ | 547.45 ± 178.97^#^ | 342.48 ± 111.81 |
| Rhu_5_ | 731.50 ± 136.27^#^ | 1353.70 ± 220.64^#^ | 49.71 ± 15.25^##^ | 48.77 ± 13.87^##^ | 763.00 ±114.31 | 425.50 ± 106.31^#^ |

All values were expressed as means ± SD. Rh_1_ – Rh_5_, rats treated with rhubarb extract at dosages of 0.21, 0.66, 2.10, 6.60 and 21.0 g/kg, respectively. ALT, alanine aminotransferase; AST, aspartate aminotransferase; TBIL, serum total bilirubin; DBIL, serum direct bilirubin; ALP, alkaline phosphatase; TBA, total bile acid. ^**^ represents *p* < 0.01 compared with the control group, ^#^ and ^##^ represents *p* < 0.05 and *p* < 0.01 compared with the RP-treated group, respectively.

1. **Parameters of PCA and OPLS-DA models and the results of OPLS-DA analysis based on data derived from ESI- mode**

**Table S5**. Parameters of PCA and OPLS-DA models

| Model No. | Model type | Groups included | Component | R^2^X | R^2^Y (cum) | Q^2^Y (cum) |
| --- | --- | --- | --- | --- | --- | --- |
| Data of ESI+ mode | | | | | | |
| M1 | PCA | Con, Rhu_4_, Model & QC | 6 | 0.505 | — | 0.181 |
| M2 | OPLS-DA | Con & Rhu_4_ | 1+1 | — | 0.995 | 0.949 |
| M3 | OPLS-DA | Rhu_4_ & Model | 1+1 | — | 0.974 | 0.708 |
| Data of ESI- mode | | | | | | |
| M4 | PCA | Con, Rhu_4_, Model & QC | 3 | 0.545 | — | 0.358 |
| M5 | OPLS-DA | Con & Rhu_4_ | 1+0 | — | 0.992 | 0.983 |
| M6 | OPLS-DA | Rhu_4_ & Model | 1+1 | — | 0.900 | 0.756 |


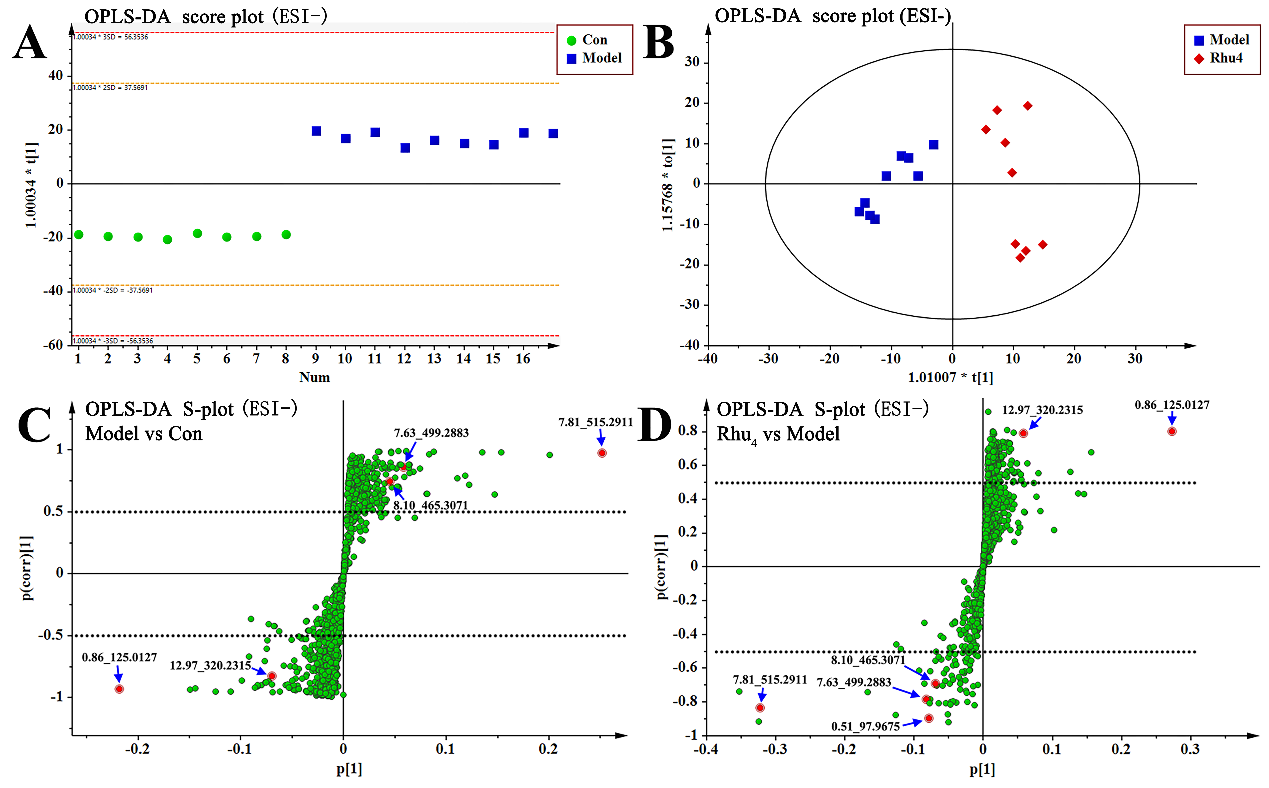


**Figure S6**. OPLS-DA analysis of data derived from ESI- mode. OPLS-DA score plots for pair-wise comparisons between Con and Model (**A**), Model and Rhu_4_ (**B**). S-plot of OPLS-DA model for Con and Model (**C**), Model and Rhu_4_ (**D**), the points in red stands for the identified biomarkers.

1. **The establishment of dose-response curve**

Dose-response curve was constructed by plotting the PCA score of first principal component and the log dose of rhubarb. According to the protocol (Miller, 2003), since the log of 0 is undefined, the model group (0 g/kg dose of rhubarb) was defined as 0.066 g/kg dose of rhubarb. A logistic distribution model with three-parameter was used for curve fitting, using the following formula.

$y=Bottom+\frac{（\mathrm{TOP}-\mathrm{Bottom}）}{1+{10}^{[log(EC50)-x]}}$ (Equation 1)

where y is the PCA score derived from the first principal component of the PCA model. Top and Bottom are plateaus in the units of the Y axis. EC_50_ is the concentration of rhubarb that gives a response half way between Bottom and Top. A goodness of fit (R^2^) value was calculated for the curve. The commercially available curve-fitting package GraphPad Prism (version 6.01 software; GraphPad, Inc., San Diego, CA) was used for the analysis.

1. **Pathway analysis of potential marker metabolites for cholestasis and rhubarb treatment**

**Table S6** Result from pathway analysis with MetaboAnalyst 3.0

| Pathway name | Total | Hits | Raw *p* | -log(*p*) | FDR | Impact |
| --- | --- | --- | --- | --- | --- | --- |
| Primary bile acid biosynthesis | 46 | 4 | 0.0006 | 7.4439 | 0.05 | 0.12 |
| Taurine and hypotaurine metabolism | 8 | 2 | 0.0022 | 6.1400 | 0.09 | 0.43 |
| Arachidonic acid metabolism | 36 | 2 | 0.0419 | 3.1732 | 0.92 | 0.00 |
| Sulfur metabolism | 5 | 1 | 0.0456 | 3.0884 | 0.92 | 0.30 |
| Arginine and proline metabolism | 44 | 2 | 0.0603 | 2.8084 | 0.98 | 0.09 |
| Sphingolipid metabolism | 21 | 1 | 0.1789 | 1.7212 | 1.00 | 0.00 |
| Glycine, serine and threonine metabolism | 32 | 1 | 0.2603 | 1.3461 | 1.00 | 0.00 |
| Tryptophan metabolism | 41 | 1 | 0.3213 | 1.1355 | 1.00 | 0.01 |
| Aminoacyl-tRNA biosynthesis | 67 | 1 | 0.4724 | 0.7500 | 1.00 | 0.00 |
| Purine metabolism | 68 | 1 | 0.4775 | 0.7391 | 1.00 | 0.00 |

Total is the total number of compounds in the pathway; the hits is the actually matched number from the user uploaded data; the raw *p* is the original *p* value calculated from the enrichment analysis; the impact is the pathway impact value calculated from pathway topology analysis.


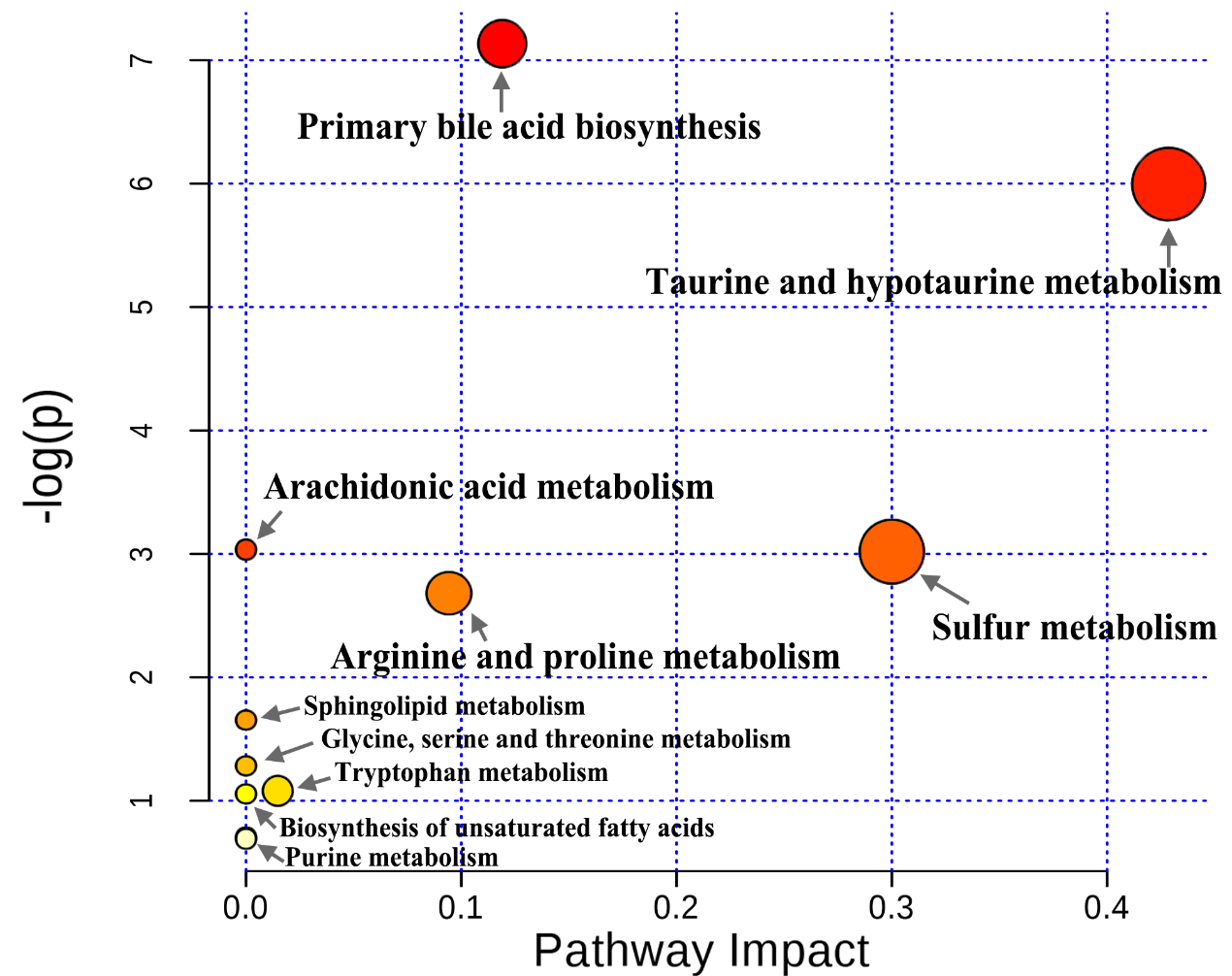


**Figure S7**. Pathway analysis of identified biomarkers derived from analysis of ESI+ and ESI- mode. Based on the identified metabolites in **Table 2**, the most perturbed pathway induced by ANIT and/or rhubarb were revealed by MetaboAnalyst 3.0 software. The pathway with high –log(*p*) and pathway impact value is greatly perturbed.

# Reference

Miller, J. (2003). "GraphPad Prism, version 4.0, Step-by-Step Examples, San Diego: Graph Pad Software". Inc).
